# Interferon-inducible protein SCOTIN interferes with HCV replication through the autolysosomal degradation of NS5A

**DOI:** 10.1038/ncomms10631

**Published:** 2016-02-12

**Authors:** Nari Kim, Min-Jung Kim, Pil Soo Sung, Yong Chul Bae, Eui-Cheol Shin, Joo-Yeon Yoo

**Affiliations:** 1 Division of Molecular and Life Sciences, Department of Life Sciences, Pohang University of Science and Technology, Pohang 790-784, Republic of Korea; 2 Laboratory of Immunology and Infectious Diseases, Graduate School of Medical Science and Engineering, KAIST, Daejeon 305-701, Republic of Korea; 3 Department of Anatomy and Neurobiology, School of Dentistry, Kyungpook National University, Daegu 41940, Republic of Korea

## Abstract

Hepatitis C virus (HCV) utilizes autophagy to promote its propagation. Here we show the autophagy-mediated suppression of HCV replication via the endoplasmic reticulum (ER) protein SCOTIN. SCOTIN overexpression inhibits HCV replication and infectious virion production in cells infected with cell culture-derived HCV. HCV nonstructural 5A (NS5A) protein, which is a critical factor for HCV RNA replication, interacts with the IFN-β-inducible protein SCOTIN, which transports NS5A to autophagosomes for degradation. Furthermore, the suppressive effect of SCOTIN on HCV replication is impaired in both ATG7-silenced cells and cells treated with autophagy or lysosomal inhibitors. SCOTIN does not affect the overall flow of autophagy; however, it is a substrate for autophagic degradation. The physical association between the transmembrane/proline-rich domain (TMPRD) of SCOTIN and Domain-II of NS5A is essential for autophagosomal trafficking and NS5A degradation. Altogether, our findings suggest that IFN-β-induced SCOTIN recruits the HCV NS5A protein to autophagosomes for degradation, thereby restricting HCV replication.

## Abstract (web summary)

Hepatitis C virus (HCV) nonstructural 5A (NS5A) protein is a critical factor for HCV RNA replication. Here the authors show that SCOTIN, an interferon beta-inducible host protein, functions to limit HCV replication by targeting viral protein NS5A for autophagosomal degradation.

## Graphical abstract

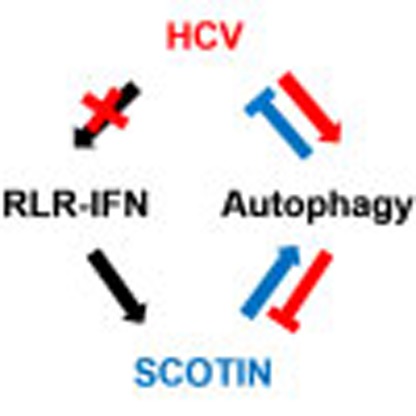

Hepatitis C virus (HCV), an RNA virus of the *Flaviviridae* family, infects more than 180 million people globally^1^. Hepatocytes are the primary target cells of HCV infection, and chronic HCV infection and the associated inflammation often lead to liver failure^2^. HCV-infected livers progressively develop hepatic fibrosis, cirrhosis and hepatocellular carcinoma if the infection is not properly treated^2^. HCV infection triggers a wide range of host cellular responses, including apoptosis, the unfolded protein response (UPR) in the endoplasmic reticulum (ER), cell cycle arrest and autophagy^3^. Although the majority of these cellular responses are activated by host cells as defenses against viral infection, these processes are often manipulated by HCV to facilitate its own survival and propagation.

HCV contains a positive-sense, single-stranded RNA genome that encodes a single polypeptide. Once the virus enters host cells, the ∼9.6-kb HCV RNA genome is translated into an ∼3,000 amino-acid polypeptide, which is then cleaved by cellular and viral proteases to produce four viral structural proteins (core, E1, E2 and p7) and six nonstructural proteins (NS2, NS3, NS4A, NS4B, NS5A and NS5B)^4^. In cells, the majority of HCV proteins are localized within or near the cytoplasmic membrane of the ER, and replication of the HCV RNA genome occurs on lipid droplets associated with an altered cytoplasmic membrane structure called the membranous web^5^. During HCV assembly, core proteins on the cytoplasmic side of the ER membrane are transferred to the luminal side with the aid of viral and cellular proteins that act to incorporate HCV proteins into virion particles and to promote their release^6^. HCV infection or the overproduction of individual HCV proteins in the ER compartment induces ER stress and the ER-stress-associated UPR^7^,^8^,^9^,^10^. Activation of the ER–UPR induces the production of reactive oxygen species and apoptosis in infected cells^7^,^9^. In addition, ER-associated degradation pathways that are triggered by ER stress control the modulation of viral protein glycosylation to limit viral production^10^. However, HCV uses these ER stress UPR pathways to deceptively modulate cellular stress responses, resulting in signals that benefit its persistent genome replication and viral translation^11^,^12^,^13^.

The ER is thought to be the major source of the autophagosomal membrane^14^. Autophagy is mediated by autophagosomes, which engulf a portion of the cytoplasm along with the target macromolecules or damaged subcellular organelles for degradation^15^. During the later stages of autophagy, autophagosomes fuse with lysosomes to form autolysosomes, in which degradation occurs^15^. Although autophagy primarily functions to maintain energy homeostasis and nutrient balance during stressful conditions such as nutrient deprivation, it plays additional diverse roles in the cell, including roles in defense against invading bacteria and cell death^16^. In contrast with the initial description of autophagy as a bulk non-selective process, recent studies have demonstrated that it uses more selective means to target and degrade intracellular pathogens beyond the detection of intrinsic protein aggregates or damaged organelles^17^,^18^. For example, to target intracellular bacteria such as *Salmonella enterica* or *Listeria monocytogenes*, the autophagy receptor protein p62 (sequestome 1 or SQSTM1) or NDP52 specifically links bacteria to LC3-containing autophagosomal membranes^19^,^20^. The p62 protein also interacts with the SIN capsid protein of the Sindbis virus, leading to the clearance of viral proteins, which protects against host cell death^21^. Furthermore, deficiencies in autophagy control result in an increased susceptibility to lethal pathogenic infections in diverse organisms, from the slime mold (*Dictyostelium*) to mice, indicating that the protective role of autophagy in pathogenic infection is evolutionarily conserved^22^,^23^.

HCV has evolved to utilize autophagy to benefit its own replication, initial translation and viral particle production^13^,^24^,^25^. HCV RNA transfection blocks lysosomal fusion with autophagosomes, the membranes of which provide replication sites for HCV^13^,^26^. Moreover, HCV-induced autophagy is required to suppress the innate antiviral responses that promote the production of interferon (IFN)-β or of interferon-stimulated genes (ISGs) and to attenuate apoptosis through mitophagy^12^,^27^,^28^. These results suggest that HCV has developed multiple ways to circumvent autophagy. However, it is unclear how HCV inhibits autolysosome formation or how it evades degradation by autolysosomes.

In the present study, we demonstrated that SCOTIN represses HCV replication through autophagy. The IFN-β-inducible ER transmembrane protein SCOTIN functions as an anti-HCV cellular factor that recruits the nonstructural 5A (NS5A) viral protein to the autophagosomal compartment, resulting in its autolysosomal degradation and the inhibition of HCV replication.

## Results

### IFN-β-inducible protein SCOTIN restricts HCV replication

**Figure 1:**
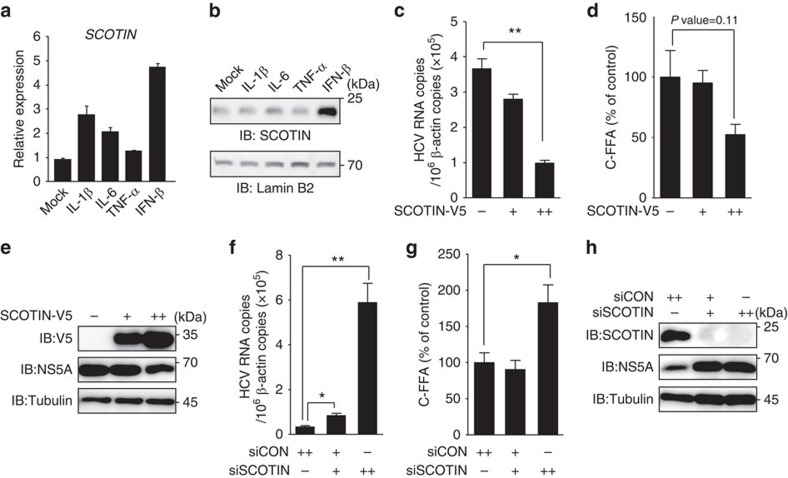
The ER protein SCOTIN inhibits HCV replication. (**a**,**b**) Huh-7 cells were incubated with IL-1β (10 ng ml^−1^), IL-6 (10 ng ml^−1^), tumour-necrosis factor (TNF)-α (10 ng ml^−1^) or IFN-β (100 U ml^−1^) for 12 h, and the expression of *SCOTIN* mRNA was measured with RT–qPCR and compared with that of *RPL32* mRNA (**a**). Endogenous SCOTIN protein levels were measured by immunoblotting. Lamin B2 was used as a loading control (**b**). The bars indicate the mean value±s.d. obtained from three experiments. (**c**–**h**) Huh-7 cells were infected with HCVcc (0.5 multiplicity of infection (MOI)) for 2 days, followed by transfection of the indicated plasmids or siRNAs for 72 h. (**c**,**f**) The intracellular HCV RNA titre was measured using reverse transcriptase–quantitative PCR (RT–qPCR) and normalized to β-actin. (**d**,**g**) Infectious HCV virions were analysed using a colorimetric focus-forming assay. The results are presented as focus-forming units (FFU) per ml of culture supernatant. (**e**,**h**) Total cell lysates were subjected to immunoblotting using the indicated antibodies. The bars indicate the mean value±s.e. obtained from triplicate experiments. The asterisks indicate the *P* values calculated using the *t*-test. *P value<0.05, **P value<0.01.

We used the following criteria to identify an unknown ISG product that restricts HCV replication: first, its expression is induced by the antiviral cytokine IFN-β; second, its known cellular compartment is the ER; and, finally, it has known biological functions related to the ER–UPR, cell cycle or cell death, which are cellular responses induced by HCV infection. Among the cellular genes whose expression is induced by type I interferon in primary hepatocytes^29^, we selected *SCOTIN*, also known as *SHISA5*, which encodes an ER membrane protein with known functions in caspase-dependent cellular apoptosis^30^, for further evaluation. Before we examined its role in HCV replication, we first determined whether its expression is induced by inflammatory cytokines in cultured hepatocytes. In Huh-7 cells treated with inflammatory cytokines, such as interleukin (IL)-1β or IL-6, or with the antiviral cytokine IFN-β, up to a two- to fivefold induction in *SCOTIN* expression was detected (Fig. 1a). However, the level of SCOTIN protein was noticeably increased only after the IFN-β treatment, indicating that its cellular protein level might be under tight control during inflammation in hepatocytes (Fig. 1b). To assess whether SCOTIN affects HCV replication, Huh-7 cells were infected with cell culture-derived HCV (HCVcc; genotype 2a) followed by overexpression of SCOTIN-V5, which encodes a V5 epitope-tagged SCOTIN protein. HCV replication and the production of infectious virions were examined by determining the HCV RNA titre and performing a focus-forming assay. SCOTIN overexpression inhibited HCV replication and virion production (Fig. 1c,d). A reduction in the levels of HCV viral proteins was separately confirmed in SCOTIN-V5-overexpressing cells (Fig. 1e). In addition, knocking down SCOTIN expression resulted in a significant degree of induction of the production of intracellular HCV and infectious virions (Fig. 1f,g). The intracellular HCV viral protein levels were consistently markedly increased in these cells (Fig. 1h). These effects of SCOTIN were examined in both Huh-luc/neo ET replicon cells containing a partial HCV replicon construct with a luciferase-reporter gene^31^ and Huh-neo-5-15 subgenomic replicon cells containing a partial HCV genome^32^. The HCV RNA level in these replicon cells was similarly decreased when SCOTIN-V5 was overexpressed and was significantly enhanced when SCOTIN expression was silenced (Supplementary Fig. 1a–c). Reduced HCV viral protein levels were also observed in SCOTIN-overexpressing Huh-neo-5-15 replicon cells (Supplementary Fig. 1d). These results suggest that the host factor SCOTIN plays inhibitory roles in the regulation of HCV replication.

### SCOTIN promotes autophagosomal degradation of NS5A

**Figure 2:**
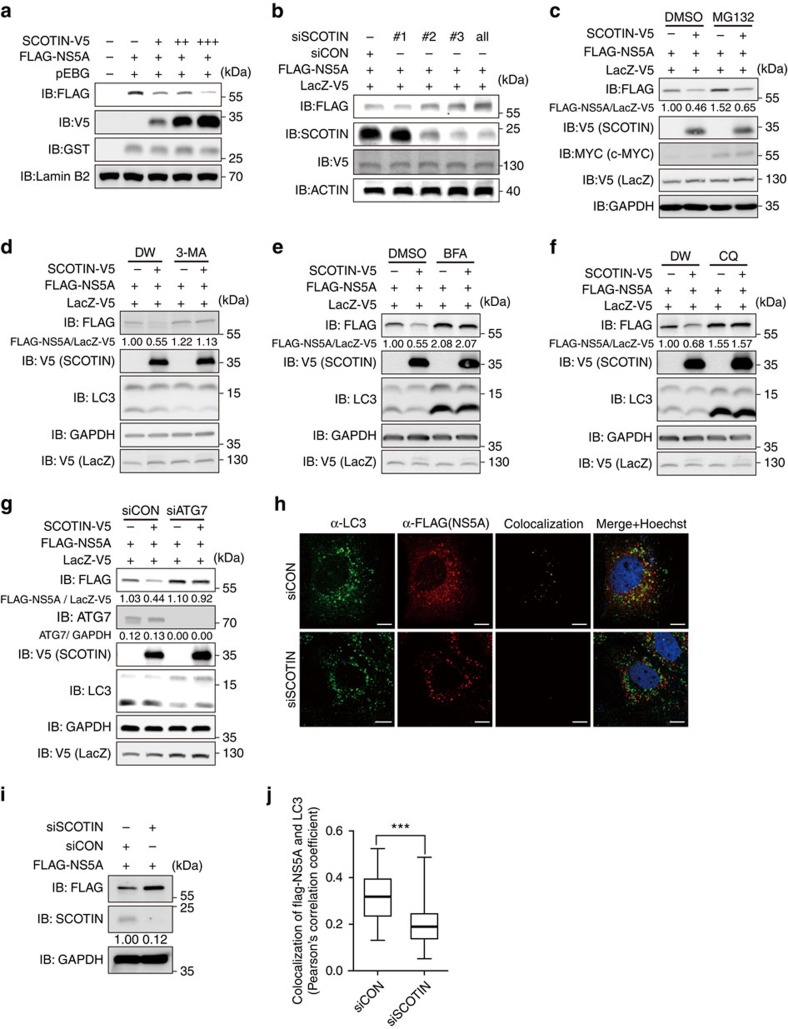
SCOTIN promotes NS5A trafficking to autophagosomes. (**a**,**b**) Huh-7 cells were transfected with the indicated plasmids or siRNAs for 48 h, and total cell lysates were subjected to immunoblotting using the indicated antibodies. A GST-expressing pEBG (**a**) or LacZ-V5 (**b**) plasmid was included to monitor transfection efficiency. (**c**–**f**) Huh-7 cells were transfected with the indicated plasmids followed by treatment with MG132 (1 μM; **c**), 3-MA (10 mM; **d**), BFA (100 μM; **e**) or CQ (50 μM; **f**) for 12 h. Total cell lysates were subjected to immunoblotting using the indicated antibodies. Distilled water (DW) was used as a control for 3-MA and CQ, and dimethylsulphoxide (DMSO) was used as a control for BFA and MG132. c-MYC was used as a positive control for MG132. The relative ratios (FLAG-NS5A/LacZ-V5) are shown based on the intensity values quantified using the Multigauge programme (Fuji Film). (**g**) Huh-7 cells transfected with the indicated plasmids along with control or ATG7 siRNA. Extracted lysates were subjected to immunoblotting using the indicated antibodies. (**c**–**g**) To determine the transfection efficiency, LacZ-V5 was co-transfected with the indicated plasmids. (**h**–**j**) Huh-7 cells were transfected with FLAG-NS5A along with control or SCOTIN siRNA, and then were treated with rapamycin (2 μM) for 6 h. (**h**) Cellular localization of endogenous LC3 (green), FLAG-NS5A (red) and the nucleus (Hoechst) was detected using confocal fluorescence microscopy. A colocalization image was obtained using the Co-localization Image J Plugin. Scale bars, 10 μm. (**i**) Total cell lysates from the same cell populations were subjected to immunoblotting using the indicated antibodies. (**j**) The extent of colocalization of FLAG-NS5A and LC3 in each cell was measured using Pearson's correlation coefficient with the JAcoP Image J Plugin. The coefficient values were plotted using a whiskers box plot. The box extends from the 25th to 75th percentiles, and the error bars depict the minimum and maximum values. Cells transfected with siCON (*N*=46) or with siSCOTIN (*N*=50) were analysed. The asterisk denotes the *P* value calculated using the *t*-test (***P value<0.001).

To investigate whether SCOTIN targets HCV proteins directly, each HCV protein was separately cloned and co-expressed with SCOTIN in Huh-7 hepatocellular carcinoma cells (Fig. 2a and Supplementary Fig. 2a). Among the HCV proteins tested, overexpression of SCOTIN specifically lowered the Flag-NS5A protein levels, but not the NS5A mRNA levels (Supplementary Fig. 2b). In addition, knockdown of endogenous SCOTIN expression enhanced the exogenous Flag-NS5A protein levels (Fig. 2b). Three different short interfering RNAs (siRNAs) specific to SCOTIN were separately examined to prevent off-target effects of siRNA from occurring. SCOTIN was observed to specifically alter the level of the NS5A protein but it did not indiscriminately affect the levels of ER proteins, as demonstrated by the lack of effect of its overexpression on the level of the ER protein GRP94 (Supplementary Fig. 1d). To demonstrate the involvement of NS5A in SCOTIN-mediated HCV regulation, the effect of BMS-790052, which has been reported to specifically inhibit NS5A (ref. 33), was assessed in control and SCOTIN siRNA-transfected Huh-neo-5-15 replicon cells. The suppressive effect of SCOTIN on HCV replication disappeared when NS5A activity was inhibited (Supplementary Fig. 2c), indicating that this protein acts to limit HCV replication, most likely through downregulating the level of NS5A.

To understand how SCOTIN controls the NS5A level, its effect on NS5A was then examined in cells treated with protein degradation inhibitors. The NS5A level was slightly increased by treatment with MG132, a 26S proteasome inhibitor, as previously reported^34^; however, the suppressive effect of SCOTIN on NS5A remained (Fig. 2c and Supplementary Fig. 2d), indicating that proteasomal degradation might not be involved in SCOTIN-mediated NS5A downregulation. In contrast, the inhibitory effect of SCOTIN on NS5A was almost completely abrogated following treatment with 3-methyladenine (3-MA), bafilomycin A1 (BFA) or chloroquine (CQ; Fig. 2d–f). 3-MA inhibits the early stages of autophagy, while BFA and CQ inhibit autolysosomal maturation and lysosomal activity, respectively^35^. As previously reported^35^, treatment of cells with 3-MA blocks the conversion of LC3-I to LC3-II, which is a molecular marker of autophagy processing, while treatment with either BFA or CQ blocks LC3-II degradation (Fig. 2d–f).

To verify the involvement of autophagy in the regulation of NS5A by SCOTIN, expression of autophagy-related genes was silenced. Knockdown of either ATG7 or ATG5, which are involved in LC3 lipidation, blocked degradation of NS5A by SCOTIN (Fig. 2g and Supplementary Fig. 2e–g). In addition, silencing of RAB7, which is required for the maturation of late autophagosomes, also prevented SCOTIN-mediated NS5A downregulation (Supplementary Fig. 2f,g). Taken together, these results indicate that SCOTIN modulates NS5A protein degradation through the autophagy–lysosome pathway.

Although NS5A is mainly localized to the membranous compartment of the ER membrane, to which it is directed by its N-terminal regions^36^, fractions of this protein, along with other HCV proteins and viral RNAs, have also been found in autophagosomes^26^. Therefore, we investigated whether SCOTIN affects the autophagosomal localization of NS5A in Huh-7 cells. LC3 protein translocates from the cytosol to autophagosomal membranes via C-terminal lipidation during autophagy and becomes arranged in a punctate pattern^37^. When SCOTIN expression was silenced in Huh-7 cells, the extent of colocalization of NS5A and LC3 was significantly reduced, suggesting that NS5A autophagosomal localization is dependent on the SCOTIN level (Fig. 2h–j). When SCOTIN-MYC and GFP (green fluorescent protein)-LC3 were transiently overexpressed, the majority of tagged SCOTIN was distributed in a punctate pattern and was colocalized with GFP-LC3-containing puncta (Supplementary Fig. 3a). In contrast, the distribution of FLAG-NS5A only partially overlapped with the GFP-LC3-containing puncta in cells transfected with FLAG-NS5A. However, when SCOTIN-MYC was co-expressed with FLAG-NS5A, a large proportion of the cells exhibiting NS5A signals displayed colocalization with GFP-LC3 puncta. Similarly, overexpressed SCOTIN and endogenously expressed NS5A protein were colocalized in Huh-neo-5-15 replicon cells, and the majority of these proteins were colocalized within GFP-LC3-containing puncta (Supplementary Fig. 3b). Taken together, these results indicate that SCOTIN might function to promote the trafficking of NS5A to the autophagosomal compartment.

To determine whether enhanced autophagosomal localization of NS5A by SCOTIN also leads to its autolysosomal localization, Huh-7 cells were co-transfected with GFP-tagged lysosomal-associated membrane protein 1 (LAMP1), which is enriched in lysosomes and late endosomes. When SCOTIN-V5 was overexpressed alone, it was observed to colocalize with a fraction of the GFP–LAMP1 structures present (Supplementary Fig. 3c, top), and when NS5A was transfected alone, it barely overlapped with these structures. However, NS5A colocalized with GFP–LAMP1 when SCOTIN was co-expressed (Supplementary Fig. 3c). Therefore, these data suggest that SCOTIN facilitates NS5A trafficking to autophagosomes, which in turn fuses with lysosomes for protein degradation.

### SCOTIN suppresses HCV replication through autophagy

**Figure 3:**
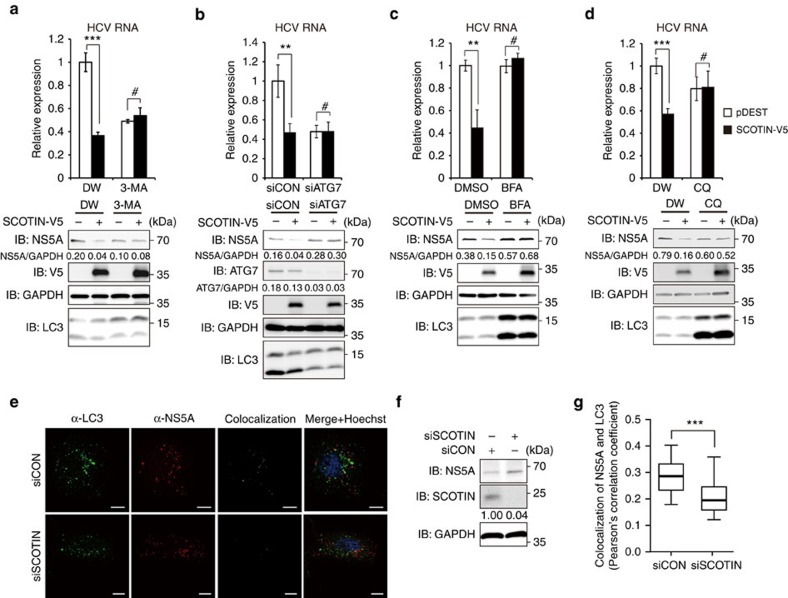
SCOTIN restricts HCV replication through autophagy-mediated protein degradation. (**a**–**d**) Huh-7 cells were transfected with the indicated plasmids or with siRNA, followed by HCVcc infection (10 MOI) for 3 days. The cells were treated with 3-MA (10 mM; **a**), BFA (100 μM; **c**) or CQ (50 μM; **d**) for 12 h before harvesting. The intracellular HCV RNA levels were measured with RT–qPCR, and total cell lysates were subjected to immunoblotting using the indicated antibodies. DW was used as a control for 3-MA and CQ, and DMSO was used as a control for BFA. The bars indicate the average value±s.d. obtained from three experiments. The asterisks denote the *P* values calculated using the *t*-test (***P value<0.001, **P value<0.01, #*P* value>0.05). (**e**–**g**) Huh-7 cells were transfected with control siRNA or SCOTIN siRNA, followed by HCVcc infection at an MOI of 10. (**e**) Cellular localization of endogenous LC3 (green), endogenous NS5A (red) and the nucleus (Hoechst) was detected using confocal fluorescence microscopy. A colocalization image was obtained using the Co-localization Image J Plugin. Scale bars, 10 μm. (**f**) Total cell lysates from the same cellular populations were subjected to immunoblotting using the indicated antibodies. (**g**) The extent of colocalization of NS5A and LC3 in each cell was measured using Pearson's correlation coefficient with the JAcoP Image J Plugin. The coefficient values were plotted using a whiskers box plot. The box extends from the 25th to 75th percentiles, and the error bar shows the minimum and maximum values. Cells transfected with siCON (*N*=37) or with siSCOTIN (*N*=45) were analysed. The asterisk denotes the *P* value calculated using the *t*-test (***P value<0.001).

To determine whether SCOTIN-mediated NS5A degradation via autophagy is an efficient mechanism for limiting HCV replication, we examined the effect of SCOTIN overexpression in Huh-7 cells infected with HCVcc at a multiplicity of infection of 10, the condition under which autophagy was induced in previous studies^12^,^24^,^38^. When the early stage of the autophagy process was blocked by treatment with 3-MA or by ATG7 knockdown, HCV replication was inhibited, as previously reported^13^,^27^,^39^, and the restriction of HCV replication by SCOTIN overexpression disappeared (Fig. 3a,b). These results indicate that the early stage of autophagy is required for the restriction of HCV replication by SCOTIN overexpression. In contrast, treatment with BFA or CQ had mild or no significant effects on HCV replication. These results can be explained by the counteracting activities of BFA and CQ on HCV replication. BFA and CQ treatment results in the accumulation of early autophagosomes^40^,^41^, which are used as replication sites for HCV^26^. At the same time, CQ treatment or a Rab7 knockdown augmented the expression of interferon-β after HCV infection and reduced the level of viral RNA^12^. The complicated effects of BFA and CQ on HCV replication may be reflected in our system. Nonetheless, BFA and CQ treatments abrogated the inhibitory effect of SCOTIN on HCV replication (Fig. 3c,d), indicating that autolysosomal activity is required for the SCOTIN-mediated restriction of HCV replication. Furthermore, the level of colocalization of NS5A with LC3-containing puncta was reduced in the SCOTIN-silenced cells (Fig. 3e–g). Taken together, these data suggest that autophagy or lysosomal activity is required for the SCOTIN-mediated downregulation of HCV replication and that SCOTIN and autophagy activity are both required for the suppression of HCV replication.

### SCOTIN does not directly alter the general autophagy process

**Figure 4:**
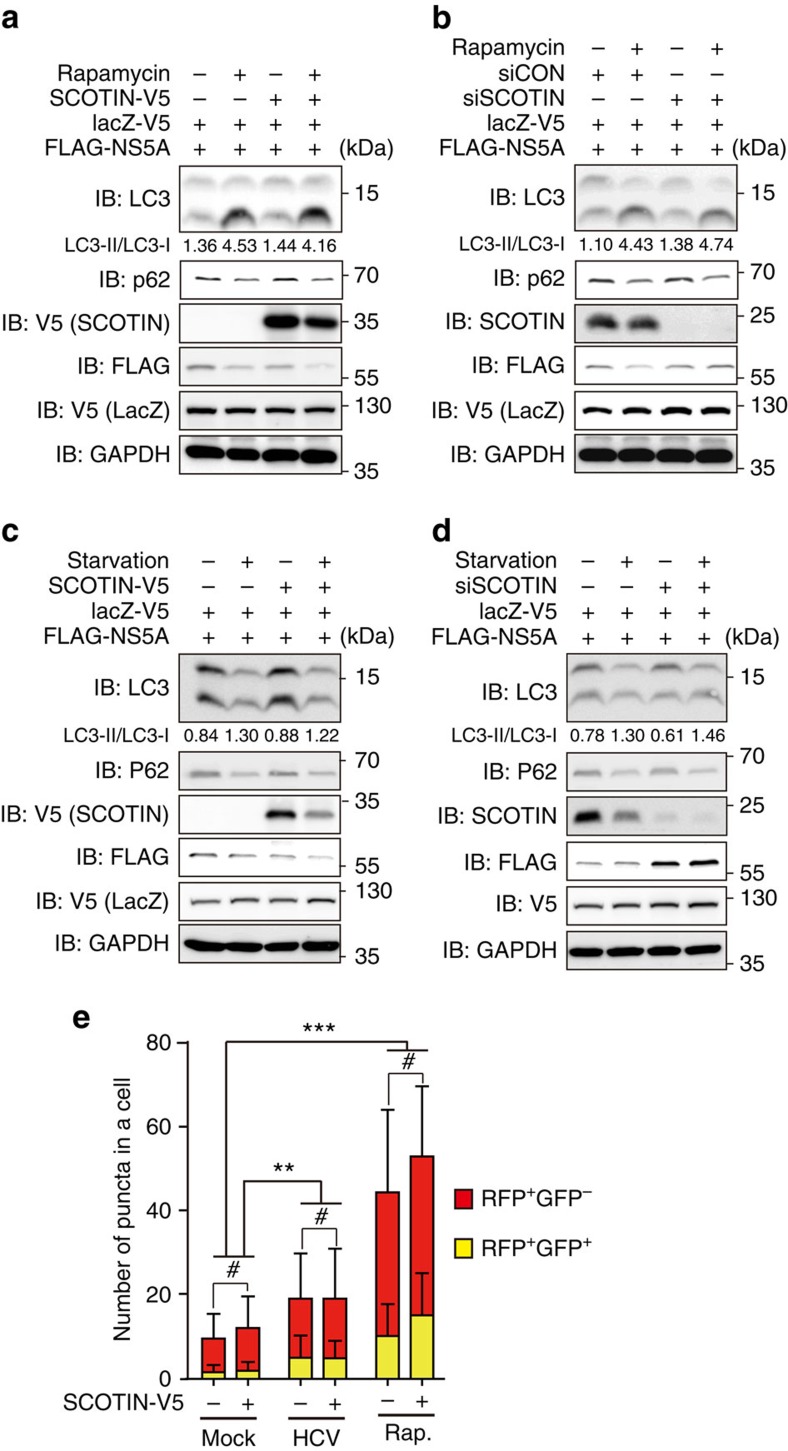
SCOTIN does not alter the overall autophagy flux. (**a**–**d**) Huh-7 cells were transfected with the indicated plasmids and/or siRNAs, followed by treatment with mock (DMSO; **a**,**b**), rapamycin (2 μM; **a**,**b**) or starvation media (**c**,**d**) for 6 h. Total cell lysates (20 μg) were subjected to immunoblotting. To monitor transfection efficiency, LacZ-V5 was co-transfected. The conversion ratio (LC3-II/LC3-I) is shown to be based on the relative signal intensities quantified using the Multigauge programme (Fuji Film). (**e**) Huh-7 cells stably expressing mRFP-GFP-LC3 were transfected with the indicated plasmid, followed by treatment with DMSO, rapamycin (2 μM, 6 h) or HCV infection (10 MOI, 72 h). The numbers of RFP^+^GFP^+^ or RFP^+^GFP^-^ puncta in each cell (*N*=100, except for SCOTIN-V5 with rapamycin: *N*=92) were counted using the Green and Red Puncta Colocalization Macro of Image J. The bars indicate the mean value±s.d. The asterisks indicate the *P* values calculated using the *t*-test. **P value<0.01, ***P value<0.001, # *P* value>0.05 (not significant).

SCOTIN degrades HCV NS5A through autophagy; therefore, we examined whether SCOTIN directly controls the overall autophagy process as a component of the autophagy machinery or whether it acts as a receptor that transports NS5A to the autophagosomal compartment. To determine whether SCOTIN is involved in autophagosome formation, the processing of cytosolic LC3-I protein to the modified autophagosomal LC3-II form was examined in SCOTIN-silenced or -overexpressing cells. However, neither steady-state nor rapamycin-induced autophagy were significantly altered in the SCOTIN-V5-overexpressing or SCOTIN-silenced cells (Fig. 4a,b). Similarly, FLAG-NS5A overexpression alone did not alter the level of LC3-II/LC3-I (Supplementary Fig. 4a). We also examined the effect of SCOTIN on the abundance of the autophagy substrate p62 protein. This protein is continuously degraded by autophagy, and its cellular level increases when autophagy is blocked^42^. Therefore, we expected that the p62 level might be altered by manipulation of SCOTIN expression if it directly controls the efficiency of the overall autophagy process. Although the p62 level was reduced by the activation of autophagy in rapamycin-treated cells, neither SCOTIN overexpression nor its knockdown considerably altered this level (Fig. 4a,b). We next examined the effect of SCOTIN on starvation-induced autophagy. Although serum starvation successfully induced autophagy as previously reported^43^, neither SCOTIN overexpression nor its knockdown significantly affected the relative conversion ratio of LC3-II/LC3-I or the abundance of p62 (Fig. 4c,d).

Finally, we examined whether SCOTIN alters the formation or maturation of autophagosomes using a tandem monomeric red fluorescent protein (mRFP)–GFP–LC3 protein-expressing construct (ptf-LC3). The autophagic flux can be measured by analysing mRFP^+/^GFP^+^ and mRFP^+^/GFP^−^ puncta in each cell because both the GFP and mRFP signals are detectable in early autophagosomes, while the GFP signal is quenched and only the mRFP signal remains in the acidic lysosome compartment^44^. For this purpose, we counted the numbers of mRFP^+^/GFP^+^ and mRFP^+^/GFP^−^ puncta in individual cells stably expressing mRFP–GFP-LC3 (Fig. 4e). Enhanced numbers of mRFP^+^/GFP^+^ and mRFP^+^/GFP^−^ were detected after rapamycin treatment or HCV infection (Fig. 4e and Supplementary Fig. 4b). However, the numbers of both types of puncta were not significantly altered by SCOTIN overexpression in basal, rapamycin-treated or HCV-infected cells (Fig. 4e and Supplementary Fig. 4b).

### SCOTIN physically interacts with NS5A to control degradation

We next examined whether SCOTIN accompanies NS5A to the autophagosomal compartment for degradation. If this action occurs, then SCOTIN must fulfill the following criteria: it must physically interact with NS5A and it must be degraded through the autophagy process.

**Figure 5:**
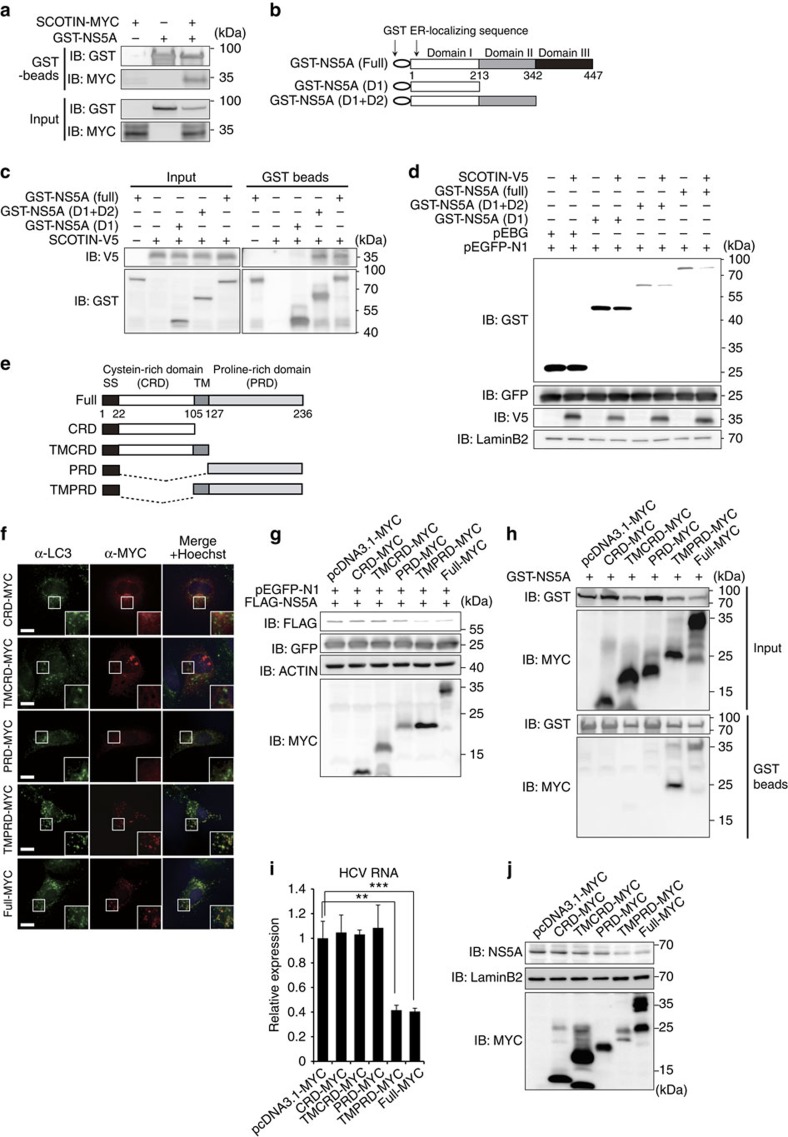
Physical interaction between NS5A and SCOTIN is required for control of degradation. (**a**) HEK293 cells were transfected with GST-NS5A along with an empty (pcDNA3.1-MYC) or SCOTIN-MYC plasmid for 48 h. GST-NS5A was pulled down from total cell lysates using glutathione-Sepharose beads, and the interacting proteins were analysed by immunoblotting. (**b**) Schematic representation of GST-tagged NS5A deletion constructs. (**c**) HEK293 cells were transfected with the indicated plasmids and subjected to a GST pulldown assay. (**d**) Huh-7 cells were transfected with the indicated plasmids for 48 h, followed by immunoblotting analysis using the indicated antibodies. EGFP was used to monitor transfection efficiency. (**e**) An illustration of the truncated SCOTIN constructs is shown. (**f**) Huh-7 cells were transfected with the indicated SCOTIN mutant constructs, and immunofluorescence analysis was performed using LC3 and MYC antibodies, followed by Hoechst staining. Cellular localization of LC3 (green), MYC (red) and the nucleus (blue) was determined using fluorescence microscopy. Representative images are shown. Higher-magnification images are shown in the right corner. Scale bar, 10 μm. (**g**) Western blot analysis of Huh-7 cells transfected with the indicated plasmids. EGFP-N1 was co-transfected to monitor transfection efficiency. (**h**) HEK293 cells were transfected with GST-NS5A along with the indicated plasmids for 48 h. Total cell lysates were incubated with glutathione-Sepharose beads, and the interacting proteins were analysed by immunoblotting. (**i**,**j**) Huh-7 cells were transfected with the indicated plasmids followed by HCVcc infection (10 MOI) for 3 days before harvesting. The intracellular HCV RNA levels were determined using RT–qPCR (**i**), and total cell lysates were subjected to immunoblotting using the indicated antibodies (**j**). The bars indicate the mean value±s.d. obtained from triplicate experiments. The asterisks indicate the *P* values calculated using the *t*-test. **P value<0.01, ***P value<0.001.

We first determined whether SCOTIN physically interacts with NS5A. To this end, cells were transiently transfected with a glutathione *S*-transferase (GST)-tagged NS5A plasmid and SCOTIN-MYC, and the physical interactions between NS5A and SCOTIN were examined using a GST pulldown assay (Fig. 5a). SCOTIN proteins were successfully precipitated with GST-NS5A proteins, suggesting that they physically interacted. Binding of NS5A with endogenous SCOTIN was also observed in Huh-neo-5-15 subgenomic replicon cells (Supplementary Fig. 5a). Next, we assessed whether a physical interaction was required for SCOTIN-mediated control of the NS5A protein level. For this purpose, we generated a series of GST-NS5A deletion constructs, and the interaction domain necessary for the NS5A–SCOTIN interaction was mapped (Fig. 5b). The NS5A protein is composed of at least three distinct functional regions. Domain I (1–213) contains the N-terminal amphipathic α-helix, which directs NS5A localization to the ER-derived membrane and zinc-binding motifs, whereas Domains II (250–342) and III (356–447) contain an IFN sensitivity-determining region and a nuclear localization signal, respectively^45^. SCOTIN-V5 was overexpressed along with a GST-tagged full-length (Full) or truncated NS5A construct, and a GST pulldown assay was performed to assess their interactions (Fig. 5c). GST-NS5A (D1+D2) and GST-NS5A (Full) exhibited similar levels of interaction with SCOTIN; however, the GST-NS5A (D1) mutant failed to interact with this protein, suggesting that Domain-II of NS5A is necessary for this interaction. We then assessed the SCOTIN-mediated degradation of full-length and truncated NS5A proteins (Fig. 5d). Consistent with the physical interaction data, SCOTIN overexpression resulted in substantial reductions in the levels of GST-NS5A (D1+D2) and GST-NS5A (Full) proteins, although no decrease was observed in the level of GST alone or of GST-NS5A (D1). Taken together, these results suggest that physical interactions between NS5A and SCOTIN are required for controlling NS5A degradation.

To map the SCOTIN domain involved in NS5A degradation, a series of SCOTIN deletion constructs was generated and was transiently transfected into Huh-7 cells (Fig. 5e). SCOTIN has the following three distinct structural domains along with an N-terminal signal sequence: a cysteine-rich domain (CRD), which localizes to the ER lumen, a single transmembrane domain and a C-terminal proline-rich domain (PRD), which faces the cytosolic side^30^,^46^. All of the generated mutant SCOTIN proteins, except for SCOTIN (TMCRD), mainly colocalized with GRP94, an ER marker protein (Supplementary Fig. 5b). The full-length and SCOTIN (transmembrane/proline-rich domain (TMPRD)) proteins were observed as punctate signals that colocalized with LC3-containing puncta in rapamycin-treated cells (Fig. 5f). In contrast, the other SCOTIN mutants exhibited distinct localization patterns relative to the LC3 puncta. The SCOTIN (CRD) mutant protein was localized near the perinuclear region, whereas the SCOTIN (PRD) mutant protein displayed a diffuse localization pattern. We also quantified the degree of colocalization of SCOTIN with LC3 puncta by counting the number of cells that exhibited co-staining (Supplementary Fig. 5c). Only the SCOTIN (TMPRD) protein colocalized with the autophagosomal compartment at a level comparable to that of the full-length SCOTIN protein.

Because SCOTIN promoted the degradation of NS5A in an autophagy-dependent manner, we next examined whether the autophagosomal localization of SCOTIN is critical for its interaction with NS5A and for degradation control. We first examined the NS5A levels in full-length and mutant SCOTIN-transfected cells (Fig. 5g). The NS5A levels were considerably decreased in cells expressing full-length SCOTIN or SCOTIN (TMPRD) but not in those expressing SCOTIN (CRD), SCOTIN (TMCRD) or SCOTIN (PRD). Furthermore, physical interaction with NS5A was detected only for the full-length SCOTIN and SCOTIN (TMPRD) constructs (Fig. 5h). The suppressive effect of SCOTIN (TMPRD) on HCV replication was consistently observed in HCVcc-infected cells, and this effect was comparable to that of full-length SCOTIN, as determined by the observed levels of intracellular HCV RNA and endogenous NS5A (Fig. 5i,j). Taken together, these results indicate that the TMPRD domain of SCOTIN is needed for autophagic trafficking and for the control of NS5A protein degradation, thereby restricting HCV replication.

### SCOTIN is degraded via HCV-induced autophagy

**Figure 6:**
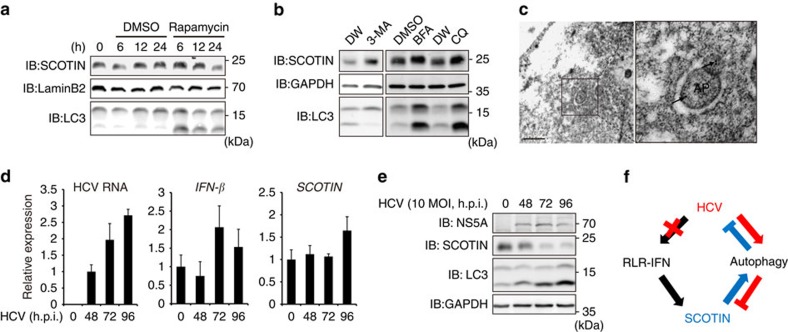
SCOTIN is degraded via HCV-induced autophagy. (**a**,**b**) Huh-7 cells were treated with rapamycin (2 μM) for the indicated durations (**a**) or with 3-MA (10 mM), BFA (100 μM) or CQ (50 μM) for 12 h (**b**). Total cell lysates were subjected to immunoblotting using the indicated antibodies. (**c**) Electron microscopic immunogold staining for the SCOTIN protein, showing its localization in autophagosomes (APs). Scale bar, 0.5 μm. Higher-magnification images are shown on the right. The gold particles (30 nm, indicated by the arrows) show the SCOTIN protein in APs. (**d**,**e**) Huh-7 cells were infected with HCVcc (10 MOI) for the indicated durations. (**d**) The expression levels of *IFN-β, SCOTIN* and HCV RNA were determined using RT–qPCR and were compared with those of *RPL32* RNA. The relative HCV RNA levels shown were determined by comparison with that of the RNA of cells infected with HCV for 48 h, which was set to 1. The relative levels of *IFN-β* and *SCOTIN* RNA were determined by comparison with their levels in control cells, which were set to 1. The bars indicate the mean value±s.d. obtained from triplicate experiments. (**e**) Total cell lysates were subjected to immunoblotting using the indicated antibodies. (**f**) Schematic illustration of the proposed mechanism.

We next examined whether SCOTIN is degraded through the autophagy-lysosomal pathway. The abundance of endogenous SCOTIN decreased over time in the rapamycin- and starvation-induced Huh-7 cells (Fig. 6a and Supplementary Fig. 5d). In addition, considerable SCOTIN accumulation was observed in cells treated with an autophagy or lysosome inhibitor (3-MA, BFA or CQ; Fig. 6b). Similar results were obtained using exogenous SCOTIN-V5 protein (Supplementary Fig. 5e). To corroborate this finding, endogenous SCOTIN was detected in autophagosomes by immunogold labelling electron microscopy (Fig. 5c). These results indicate that the SCOTIN level might be maintained by autophagy-mediated degradation processes.

Thus far, we have demonstrated that SCOTIN targets the NS5A protein to autophagosomes for degradation. However, it is also known that HCV induces autophagy to benefit its replication^47^. Therefore, we examined the effects of HCV infection on *SCOTIN* expression. During HCVcc infection, no significant *IFN-β* expression was observed; hence, the induction of *SCOTIN* mRNA expression was not detected (Fig. 6d). Although primary human hepatocytes and Huh-7-TLR3 cells produce type I and type III IFN after being infected with HCVcc at a high titre, the Huh-7 cell line is known to be a poor producer of type I and III IFNs after HCVcc infection^48^. Interestingly, however, the level of endogenous SCOTIN was dramatically decreased (Fig. 6e). HCVcc infection induced autophagy, as demonstrated by the enrichment of LC3-II, and SCOTIN was detected within the autophagosomal compartment after HCVcc infection (Supplementary Fig. 5f). Therefore, these results indicate that HCV eliminates SCOTIN protein in the infected host cells, presumably via enhancing the autophagy process.

## Discussion

In this report, we demonstrate that an IFN-β-inducible host factor, SCOTIN, contributes to suppressing HCV replication via the autolysosomal degradation of the HCV NS5A protein. SCOTIN is an ER transmembrane protein that physically associates with NS5A and is also a substrate for autophagy-mediated degradation. Similar to the role of p62 autophagy receptor in the autophagy-mediated clearance of intracellular viruses and bacteria, SCOTIN recruits NS5A to LC3-II-containing autophagosomal compartments through physical interactions. These physical interactions and the colocalization of SCOTIN and NS5A in autophagosomes are critical for controlling the autophagy-mediated proteolytic degradation of the NS5A viral protein and HCV replication.

In the present work, we propose that SCOTIN is one of the ISGs that interferes with HCV replication. Type I IFNs exhibit most of their antiviral activities via hundreds of ISGs, which involve various proteins, including enzymes, transcription factors, heat-shock proteins and apoptotic proteins^49^. SCOTIN expression was induced in Huh-neo-5-15 HCV subgenomic replicon cells treated with IFN-β, and the suppressive effect of IFN-β on HCV replication was reduced when SCOTIN expression was silenced (Supplementary Fig. 6a–c). These results indicate that SCOTIN acts as one of the ISGs that contribute to the antiviral activity of interferon-β that is directed against HCV.

We have demonstrated that the host factor SCOTIN transports NS5A to autophagosomes and promotes its autolysosomal degradation. NS5A produced during an HCV infection can be cleared by autophagy if autolysosomes are active and a sufficient SCOTIN level is present. However, no significant increase in *SCOTIN* and *IFN-β* mRNA was observed after HCV infection (Fig. 6d), which might be caused by the inhibition of RIG-I-like-receptor-mediated IFN-β induction^50^. In addition, a diminished SCOTIN level was detected after HCV infection when autophagy was induced (Fig. 6e). Therefore, it is highly likely that autophagy induced by HCV facilitates the degradation of this protein. These findings demonstrate a novel scenario of competition between host and viral proteins that involves the degradative processes of autophagy (Fig. 6f). If the steady-state SCOTIN level is sufficiently high to overpower HCV at earlier stages of infection, then this protein recruits NS5A to the autolysosomal compartment for degradation and inhibits viral replication. However, if the infected HCV overwhelms SCOTIN at earlier stages, then the virus induces autophagy at a level sufficient to destroy this protein and evade the host antiviral response. This scenario somewhat resembles the cro-cII protein degradation controls that determine the fate of *Escherichia coli* infected with lambda bacteriophage^50^. As the cellular level of the cII protein that infected lambda encounters is the critical parameter that determines the lysogenic or lytic cycle, the cellular level of SCOTIN at the boundary of HCV infection might play key roles in the tug-of-war between the virus and host. In that sense, induction of SCOTIN production by paracrine interferon-β in non-infected, neighbouring cells might confer immunity against infection. Our model therefore suggests that SCOTIN might be more effective in controlling the infection at an earlier stage than it is during the pre-established chronic infection state.

Although autophagy is known to facilitate HCV replication, there are some controversies regarding the controlling mechanism of autophagy by HCV. HCV infection has been proposed to cause the incomplete maturation of autophagic vesicles to prevent autophagic protein degradation^13^, and HCV has been found to utilize enriched autophagosomal membranes for its replication^26^. However, other reports suggested that HCV induces complete autophagy processes and that full autolysosomal activity is required for the repression of the innate immune response^12^,^27^. In the present study, we also observed that HCVcc infection induced autophagy accompanied by lysosomal activity (Fig. 4e). Because 3-MA treatment or ATG7 knockdown resulted in a decrease in the level of HCV RNA in the HCVcc experiments (Fig. 3a,b), it is likely that HCV-induced autophagy regulation also occurred in our HCVcc infection study. Nevertheless, our data suggest that HCV can be cleared by autophagy processes when a sufficient amount of SCOTIN protein is present. At this moment, it is not clear whether the general autophagy process is utilized or whether selective autophagy is required for the clearance of HCV by SCOTIN.

*SCOTIN* was first identified as a direct target gene of the p53 transcription factor^30^. The expression is specifically induced under p53-activating, DNA-damaging conditions, such as ultraviolet irradiation or actinomycin D treatment, and it has been suggested to be involved in caspase-dependent apoptosis^30^. SCOTIN has also been reported to physically interact with the calcium-binding protein ALG-2, which is known to interact with the AIP1, ALIX and TSG101 proteins, which control intracellular vesicle trafficking and apoptosis^46^. Complicated crosstalk occurs between autophagy and apoptosis^51^; therefore, we considered the possible involvement of SCOTIN-mediated apoptosis in the regulation of HCV replication. However, the IFN-β-inducible expression of SCOTIN was independent of p53, as similar levels of SCOTIN protein induction were observed in IFN-β-stimulated HCT116 p53 wild-type and HCT116 p53 null cells (Supplementary Fig. 6d). The basal SCOTIN level was relatively lower in p53 null cells, suggesting that p53 might function to maintain a steady-state SCOTIN level. We further determined whether SCOTIN overexpression alone induces apoptosis. In the absence of DNA damage stress signals, however, the basal level of apoptosis was not considerably increased by SCOTIN overexpression alone, as determined by the proteolytic cleavage of PARP-1 proteins (Supplementary Fig. 1d). Furthermore, the functional domains that control p53-dependent apoptosis and autophagosomal degradation of HCV NS5A are distinct. The CRD of SCOTIN is required for promoting p53-dependent pro-apoptotic activity^30^, and the TMPRD is sufficient for localization to autophagosomes and degradation of the NS5A protein (Fig. 5f,g). These results suggest that SCOTIN is involved in at least two distinct cellular processes, namely apoptosis and autophagy.

Among the viral proteins produced by HCV, NS5A is the most enigmatic. It does not demonstrate any intrinsic enzymatic activity, although it interacts with many cellular proteins, including PKR, TRAF2, Grb2 and Bax (ref. 45). NS5A is critical for HCV RNA replication^45^. Because of its multiple functions in HCV replication and assembly and in modulating cellular pathways, NS5A inhibitors are considered to be the most potent anti-HCV drugs in development, and compounds such as daclatasvir have been reported to possess pan-genotypic activity and high potency in clinical trials^52^. Among the many postulated roles of NS5A, its localization to the ER or to ER-derived compartments via its N-terminal amphipathic alpha-helical domain is critical because disruption of its association with the ER membrane directly alters HCV replication^53^. There is controversy regarding NS5A trafficking to ER-derived autophagosomes. NS5A, along with other HCV proteins and RNA, is localized to autophagosomes, where HCV uses the membrane as a platform for replication^26^. Other groups have also proposed that the localization of NS5A is distinct from that of GFP-LC3 puncta, and that components of the autophagy pathway act as proviral factors to assist with the translation of incoming HCV RNA or with viral particle production^24^,^25^.

In this report, we demonstrated that alterations in the steady-state SCOTIN level did not affect autophagy progression (Fig. 4). Instead, its level was reduced by the activation of autophagy and increased following treatment with an autophagy or lysosome inhibitor (Fig. 6a,b). These data indicate that SCOTIN may be a substrate of the autophagic proteolysis pathway itself rather than a universal component of autophagosomal machinery. Various autophagy receptors are involved in the selective sequestration of diverse endogenous or exogenous target substrates to autophagosomes. These autophagy receptor proteins, including p62, NBR1, BNIP3, NIX, NDP52 and OPTN, are capable of tethering autophagy substrates to autophagosomal membrane via unique cargo-recognizing domains, as well as LC3/GABARAP-interacting regions that facilitate the association of substrates with autophagosomal membranes^17^. In addition, these receptor proteins are destroyed by autophagosomal degradation. We observed that SCOTIN shares many features with these receptor proteins. It colocalizes with LC3 in autophagosomes, selectively binds to NS5A, an autophagic substrate, and is degraded via autophagy. However, the mechanism of the selective localization of SCOTIN to autophagosomes is unclear, as is whether there is a known autophagy receptor that is specifically involved. Therefore, elucidation of the regulatory mechanism of SCOTIN recruitment to autophagosomes would be of great interest in future studies.

## Methods

### Cell culture and reagents

Huh-7 (a gift from S.K. Jang, POSTECH, Republic of Korea) and HEK293 (obtained from American Type Culture Collection) cells were grown in DMEM (Welgene) supplemented with 10% fetal bovine serum (Hyclone). Huh-luc/neo-ET and Huh-neo-5-15 HCV subgenomic replicon cells (a gift from Bartenschlager, R., University of Heidelberg, Germany) were grown in DMEM media containing G418 (600 μg ml^−1^; Calbiochem). Huh-7 stable cells expressing mRFP-GFP-LC3 (ptf-LC3, a gift from Tamotsu Yoshimori, Addgene plasmid #21074) were selected using G418 (1.2 mg ml^−1^). Plasmid and siRNA transfections were performed with Lipofectamine 2000 (Invitrogen) in accordance with the manufacturer's instructions. For stimulation, the cells were treated with IL-1β (10 ng ml^−1^, R&D Systems), IL-6 with IL-6sR (each 10 ng ml^−1^, R&D Systems), tumour-necrosis factor-α (10 ng ml^−1^, R&D systems) and IFN-β (100 U ml^−1^, R&D systems) for 12 h. To perturb autophagy, the cells were treated with rapamycin (2 μM; Sigma-Aldrich) and incubated in low-glucose DMEM (purchased from Welgene), 3-MA (10 mM; Sigma-Aldrich), BFA (100 μM; Sigma-Aldrich) or CQ (50 μM; Sigma-Aldrich) for the indicated durations. To induce starvation, starvation media (140 mM NaCl, 1 mM CaCl_2_, 1 mM MgCl_2_, 5 mM glucose, 20 mM HEPES, pH 7.4) was applied to cells for the indicated duration after washing the cells twice using PBS, as previously reported^43^. BMS-790052 (1 nM, S1482, Selleckchem) was used to inhibit NS5A protein. For the knockdown experiments, control, ATG7, ATG5, RAB7 and SCOTIN siRNAs were purchased from GenePharma; their sequences are listed in Supplementary Table 1.

### Plasmids

The HCV-E2, NS3, NS4, NS5A and NS5B plasmids have been previously described^54^. The plasmid pHAGE-Ubc-LAMP1-GFP was a generous gift from Y.M. Kim (POSTECH, Republic of Korea). For GFP-LC3 expression plasmid, CDS coding LC3B protein was amplified using cDNA produced from HepG2 cells and ligated with EGFP-C3 plasmid digested with XhoI and KpnI. Scotin isoform1 CDS was amplified using cDNA from lipopolysaccharide-treated mouse liver, and then cloned into pDEST-V5 plasmids digested with SpeI and NheI. SCOTIN deletion mutations for PRD and TMPRD were created via the QuickChange Site-Directed Mutagenesis Kit (Stratagene). SCOTIN deletion mutation for CRD and TMCRD was generated using primers described in Supplementary Table 2. Full-length or deleted NS5A sequence was amplified from pCMV-FLAG-NS5A plasmid using primers described in Supplementary Table 2, and they were cloned into mammalian GST expression vector pEBG.

### Cell lysis and immunoblot analysis

Cell pellets were lysed with lysis buffer (150 mM NaCl, 1% Triton X-100, 25 mM Tris, pH 7.5, 0.5% deoxycholic acid, 0.1% SDS, 1 mM DTT, 2 μg ml^−1^ pepstatin, 0.1 mg ml^−1^ phenylmethyl sulphonyl fluoride, 5 μg ml^−1^ aprotinin, 5 μg ml^−1^ leupeptin and 1 mM benzamidine), and the lysate was resolved on an SDS–PAGE gel and transferred to a nitrocellulose membrane (Bio-Rad). For immunoblotting analysis, the membranes were probed with primary antibodies, followed by horseradish peroxidase (HRP)-conjugated secondary antibodies, and they were visualized using an LAS 4000 image Reader (Fuji Film, Japan) after ECL treatment (Pierce).

### Immunoprecipitation and GST pulldown

For immunoprecipitation, 1 mg of total cell lysate was incubated with mouse normal IgG or an NS5A antibody for 12 h at 4 °C, followed by incubation with protein A/G agarose beads (Calbiochem) for 2 h. For the GST pulldown experiments, 1 mg of cell lysate was incubated with 20 μl of glutathione-Sepharose beads (Amersham) at 4 °C overnight. The beads were then washed with lysis buffer three times, and proteins were eluted from the beads by incubation with Laemmli sample buffer (Bio-Rad) supplemented with 5% β-mercaptoethanol at 95 °C for 10 min. For the immunoprecipitation and GST pulldown assays, 1/20th of the total cell lysate volume was loaded as the input sample.

### RNA extraction and RT–qPCR analysis

Total RNA was extracted using RNAiso Plus (Takara), and 1 μg of RNA was reverse-transcribed into cDNA using random primers and an ImProm-II Reverse Transcription Kit (Promega). The synthesized cDNA was analysed by quantitative real-time PCR using 2X SYBR premix (Takara) and a StepOnePlus real-time PCR system (ABI). The oligomer sequences are listed in Supplementary Table 3. The level of intracellular HCV RNA was quantified using primers described in Supplementary Table 3 and standardized to a β-actin Taqman primer (appliedbiosystems, 4310881E)^55^.

### Luciferase assay

Huh-luc/neo-ET HCV subgenomic replicon cells were lysed with passive lysis buffer (Promega), and luciferase acitivity was analysed using a Dual Luciferase Assay Kit (Promega) after protein quantitation using the Bradford assay to adjust for the amount of cell lysate.

### Immunofluorescence staining and analysis

Cells were grown on poly-D-lysine-coated glass cover slips and fixed with 4% paraformaldehyde in PBS before immunostaining. The cells were then permeabilized with 0.2% Triton X-100 and blocked with 3% goat serum and 5% bovine serum albumin. Next, they were incubated with the indicated antibody at 4 °C overnight. After washing with PBST, the cells were incubated with Alexa Fluor 488-, Alexa Fluor 405- or Alexa 568-conjugated IgG (Invitrogen) for 2 h at 4 °C. Slides were incubated with Hoechst 33258 (Sigma-Aldrich) during the PBST washing step, and then mounted and analysed using an epifluorescence microscope (Zeiss) or FV1000 confocal microscope (Olympus). For LC3 staining, the cells were permeabilized with 100% methanol for 10 min at −20 °C and were blocked with 5% goat serum and 0.3% Triton X-100 in PBS. Next, they were incubated with the indicated antibody in 1% bovine serum albumin (BSA) and 0.3% Triton X-100 in PBS at 4 °C overnight. After washing with PBS, the cells were incubated with the secondary antibodies described above in PBS containing 1% BSA and 0.3% Triton X-100 for 1 h at room temperature. For endogenous NS5A staining, all of the washing and antibody-incubation steps were performed in Tris-buffered saline containing 0.1% Triton X-100 (TBST) instead of PBS or PBST. Pearson's correlation coefficient was calculated using the JACoP plugin for Image J. Quantification of RFP^+^/GFP^+^ or RFP^+^/GFP^−^ puncta was performed using the Green and Red Puncta Colocalization Macro for Image J (Daniel J. Shiwarski, Ruben K. Dagda and Charleen T. Chu).

### Antibodies

The following primary antibodies were used at indicated dilution: SCOTIN (Santa Cruz Biotechnology, sc-390725, western blot (WB)—1:500), MYC (Santa Cruz Biotechnology, sc-40, WB—1:1,000), V5 (Invitrogen, R960-25, WB—1:5,000), FLAG (Sigma-Aldrich (mouse), F3165, immunofluorescence (IF)—1:200), FLAG (Santa Cruz Biotechnology (rabbit), sc-807, WB—1:2,000), ACTIN (Santa Cruz Biotechnology, sc-1616, WB—1:5,000), Lamin B2 (Santa Cruz Biotechnology, sc-6216, WB—1:5,000), α-tubulin (Sigma-Aldrich, clone B-5-1-2, WB—1:4,000), GST (Santa Cruz Biotechnology, sc-138, WB—1:1,000), GRP94 (Abcam, ab18055, IF—1:200, WB—1:1,000), PARP-1 (Santa Cruz Biotechnology, sc-8007, WB—1:1,000), LC3B (Cell signaling, #2775, WB—1:500, IF—1:100), p62 (BD Bioscience, 610832, WB—1:2,000), ATG7 (Santa Cruz Biotechnology, sc-33122, WB—1:1,000), GAPDH (Chemicon, MAB374, WB—1:25,000), SCOTIN (JC105, gift from D.P. Lane, University of Dundee, IF—1:100), HCV NS5A (Thermo Scientific, MA1-82923, used for IF staining, IF—1:100) and HCV NS5A (Virogen, 256-A, used for WB and IP, WB—1:500). The following secondary antibodies were used for immunoblot analysis: goat anti-mouse HRP-conjugated IgG (Pierce, 1858413, 1:25,000), goat anti-rabbit HRP-conjugated IgG (Pierce, 1858415, 1:25,000) and donkey anti-goat HRP-conjugated IgG (Santa Cruz Biotechnology, sc-2020, 1:5,000).

### Immunogold labelling of SCOTIN protein

Cells were fixed in 0.005% glutaraldehyde and 4% paraformaldehyde in 0.1 M phosphate buffer (pH 7.4) for 2 h at 4 °C. The cells were washed three times with phosphate buffer. For low-temperature embedding, the cells were transferred to Leica EM AFS (Leica Instruments, Vienna, Austria) and were treated as follows: the cells were dehydrated in increasing concentrations of ethanol (30, 50 and 70% at 4 °C, and 80, 95 and 100% at −20 °C). The cells were washed two times with absolute ethanol and were infiltrated with Lowicryl HM20 resin (ethylmethane sulphonate, Lowicryl HM20 embedding kit) at −20 °C: 1 h in each of 50, 75 and 100% Lowicryl HM20 and then 18 h in 100% Lowicryl HM20. The resin was polymerized using ultraviolet light (48 h each at −20 and +20 °C). Ultrathin sections were cut (65–70 nm thickness) and were collected on 300-mesh nickel grids. The grids were etched for 3 s in sodium ethanolate (a saturating concentration of NaOH dissolved in 100% ethanol at least 24 h before use). The grids were wetted in TBST (pH 7.6) and then were incubated for 20 min in a solution of 2% human serum albumin (Sigma-Aldrich, St Louis, MO) containing 0.1% Na-borohydride and 50 mM glycine. The grids were then washed with TBST and incubated for 2 h at room temperature with a rabbit anti-SCOTIN antibody (1: 50, Santa Cruz Biotechnology, sc-103188). The grids were rinsed two times each for 10 min in TBST and then were incubated for 1.5 h in goat anti-rabbit IgG conjugated to 30-nm gold particles (1:25 in TBST containing 0.05% polyethylene glycol; BBI, UK). After rinsing with distilled water, the grids were stained with uranyl acetate and lead citrate and were examined using a Hitachi H-7500 electron microscope (Tokyo, Japan). Digital images were captured using the Digital Montage software driving a charge-coupled device camera (SC1000; Gatan, Pleasanton, CA, USA) attached to an electron microscope operated at 80 kV of accelerating voltage. Substituting the primary antiserum with normal rabbit serum completely abolished the specific labelling.

### Generation and infection of JFH-1 HCVcc

The Japanese Fulminant Hepatitis-1 (JFH-1) strain (genotype 2a) of HCVcc^56^,^57^,^58^ was generated and propagated as follows^59^: Huh-7.5 cells were transfected with *in vitro*-transcribed JFH-1 RNA with DMRIE-C reagent (Invitrogen), and the culture supernatant with the highest viral titre was harvested to infect naive Huh-7.5 cells. The HCV-infected Huh-7.5 cells were passaged, and the cell culture supernatants with the highest HCV production were selected. The selected supernatants were passed through a 0.45-μm filter and frozen until use. To titrate the infectivity of JFH-1, a colorimetric focus-forming assay^60^ was performed as follows: briefly, Huh-7.5 cells were plated in 96-well, collagen-coated plate. Next day, JFH-1 HCVcc stocks were inoculated after serial dilutions. After 72 h, cells were fixed and permeabilized with 100% methanol. Cells were stained with primary antibody against HCV core and alkaline phosphatase-conjugated secondary antibody against anti-mouse IgG. After vigorous washing, BCIP/NBT chromogenic substrate was used for foci development. The number of foci was determined using an ELISpot reader and the image analysis software. Huh-7 cells were infected with JFH-1 at the indicated multiplicity of infection.

## Additional information

**How to cite this article:** Kim, N. *et al.* Interferon-inducible protein SCOTIN interferes with HCV replication through the autolysosomal degradation of NS5A. *Nat. Commun.* 7:10631 doi: 10.1038/ncomms10631 (2016).

## Supplementary Material

Supplementary Information Supplementary Figures 1-7 and Supplementary Tables 1-3

## References

[b1] Mohd Hanafiah K., Groeger J., Flaxman A. D. & Wiersma S. T. Global epidemiology of hepatitis C virus infection: new estimates of age-specific antibody to HCV seroprevalence. Hepatology 57, 1333–1342 (2013). 23172780 10.1002/hep.26141

[b2] Lavanchy D. The global burden of hepatitis C. Liver Int. 29, (Suppl 1): 74–81 (2009). 19207969 10.1111/j.1478-3231.2008.01934.x

[b3] Ke P. Y. & Chen S. S. Hepatitis C virus and cellular stress response: implications to molecular pathogenesis of liver diseases. Viruses 4, 2251–2290 (2012). 23202463 10.3390/v4102251 PMC3497051

[b4] Lindenbach B. D. & Rice C. M. Unravelling hepatitis C virus replication from genome to function. Nature 436, 933–938 (2005). 16107832 10.1038/nature04077

[b5] Scheel T. K. & Rice C. M. Understanding the hepatitis C virus life cycle paves the way for highly effective therapies. Nat. Med. 19, 837–849 (2013). 23836234 10.1038/nm.3248 PMC3984536

[b6] Jones D. M. & McLauchlan J. Hepatitis C virus: assembly and release of virus particles. J. Biol. Chem. 285, 22733–22739 (2010). 20457608 10.1074/jbc.R110.133017 PMC2906262

[b7] Benali-Furet N. L. *et al.* Hepatitis C virus core triggers apoptosis in liver cells by inducing ER stress and ER calcium depletion. Oncogene 24, 4921–4933 (2005). 15897896 10.1038/sj.onc.1208673

[b8] Merquiol E. *et al.* HCV causes chronic endoplasmic reticulum stress leading to adaptation and interference with the unfolded protein response. PLoS ONE 6, e24660 (2011). 21949742 10.1371/journal.pone.0024660 PMC3176279

[b9] Li S. *et al.* Hepatitis C virus NS4B induces unfolded protein response and endoplasmic reticulum overload response-dependent NF-kappaB activation. Virology 391, 257–264 (2009). 19628242 10.1016/j.virol.2009.06.039

[b10] Saeed M. *et al.* Role of the endoplasmic reticulum-associated degradation (ERAD) pathway in degradation of hepatitis C virus envelope proteins and production of virus particles. J. Biol. Chem. 286, 37264–37273 (2011). 21878646 10.1074/jbc.M111.259085 PMC3199473

[b11] Tardif K. D., Mori K. & Siddiqui A. Hepatitis C virus subgenomic replicons induce endoplasmic reticulum stress activating an intracellular signaling pathway. J. Virol. 76, 7453–7459 (2002). 12097557 10.1128/JVI.76.15.7453-7459.2002 PMC136367

[b12] Ke P. Y. & Chen S. S. Activation of the unfolded protein response and autophagy after hepatitis C virus infection suppresses innate antiviral immunity *in vitro*. J. Clin. Invest. 121, 37–56 (2011). 21135505 10.1172/JCI41474 PMC3007134

[b13] Sir D. *et al.* Induction of incomplete autophagic response by hepatitis C virus via the unfolded protein response. Hepatology 48, 1054–1061 (2008). 18688877 10.1002/hep.22464 PMC2562598

[b14] Tooze S. A. & Yoshimori T. The origin of the autophagosomal membrane. Nat. Cell Biol. 12, 831–835 (2010). 20811355 10.1038/ncb0910-831

[b15] Mizushima N. Autophagy: process and function. Genes Dev. 21, 2861–2873 (2007). 18006683 10.1101/gad.1599207

[b16] Mizushima N. The pleiotropic role of autophagy: from protein metabolism to bactericide. Cell Death Differ. 12, (Suppl 2): 1535–1541 (2005). 16247501 10.1038/sj.cdd.4401728

[b17] Stolz A., Ernst A. & Dikic I. Cargo recognition and trafficking in selective autophagy. Nat. Cell Biol. 16, 495–501 (2014). 24875736 10.1038/ncb2979

[b18] Levine B., Mizushima N. & Virgin H. W. Autophagy in immunity and inflammation. Nature 469, 323–335 (2011). 21248839 10.1038/nature09782 PMC3131688

[b19] Yoshikawa Y. *et al.* *Listeria monocytogenes* ActA-mediated escape from autophagic recognition. Nat. Cell Biol. 11, 1233–1240 (2009). 19749745 10.1038/ncb1967

[b20] Thurston T. L., Ryzhakov G., Bloor S., von Muhlinen N. & Randow F. The TBK1 adaptor and autophagy receptor NDP52 restricts the proliferation of ubiquitin-coated bacteria. Nat. Immunol. 10, 1215–1221 (2009). 19820708 10.1038/ni.1800

[b21] Orvedahl A. *et al.* Autophagy protects against Sindbis virus infection of the central nervous system. Cell Host Microbe 7, 115–127 (2010). 20159618 10.1016/j.chom.2010.01.007 PMC2860265

[b22] Jia K. *et al.* Autophagy genes protect against *Salmonella typhimurium* infection and mediate insulin signaling-regulated pathogen resistance. Proc. Natl Acad. Sci. USA 106, 14564–14569 (2009). 19667176 10.1073/pnas.0813319106 PMC2731839

[b23] Deretic V. & Levine B. Autophagy immunity, and microbial adaptations. Cell Host Microbe 5, 527–549 (2009). 19527881 10.1016/j.chom.2009.05.016 PMC2720763

[b24] Dreux M., Gastaminza P., Wieland S. F. & Chisari F. V. The autophagy machinery is required to initiate hepatitis C virus replication. Proc. Natl Acad. Sci. USA 106, 14046–14051 (2009). 19666601 10.1073/pnas.0907344106 PMC2729017

[b25] Tanida I. *et al.* Knockdown of autophagy-related gene decreases the production of infectious hepatitis C virus particles. Autophagy 5, 937–945 (2009). 19625776 10.4161/auto.5.7.9243

[b26] Sir D. *et al.* Replication of hepatitis C virus RNA on autophagosomal membranes. J. Biol. Chem. 287, 18036–18043 (2012). 22496373 10.1074/jbc.M111.320085 PMC3365724

[b27] Shrivastava S., Raychoudhuri A., Steele R., Ray R. & Ray R. B. Knockdown of autophagy enhances the innate immune response in hepatitis C virus-infected hepatocytes. Hepatology 53, 406–414 (2011). 21274862 10.1002/hep.24073 PMC3335751

[b28] Kim S. J. *et al.* Hepatitis C virus triggers mitochondrial fission and attenuates apoptosis to promote viral persistence. Proc. Natl Acad. Sci. USA 111, 6413–6418 (2014). 24733894 10.1073/pnas.1321114111 PMC4035934

[b29] Thomas E. *et al.* HCV infection induces a unique hepatic innate immune response associated with robust production of type III interferons. Gastroenterology 142, 978–988 (2012). 22248663 10.1053/j.gastro.2011.12.055 PMC3435150

[b30] Bourdon J. C., Renzing J., Robertson P. L., Fernandes K. N. & Lane D. P. Scotin, a novel p53-inducible proapoptotic protein located in the ER and the nuclear membrane. J. Cell Biol. 158, 235–246 (2002). 12135983 10.1083/jcb.200203006 PMC2173124

[b31] Vrolijk J. M. *et al.* A replicon-based bioassay for the measurement of interferons in patients with chronic hepatitis C. J. Virol. Methods 110, 201–209 (2003). 12798249 10.1016/s0166-0934(03)00134-4

[b32] Lohmann V. Replication of subgenomic hepatitis C virus RNAs in a hepatoma cell line. Science 285, 110–113 (1999). 10390360 10.1126/science.285.5424.110

[b33] Gao M. *et al.* Chemical genetics strategy identifies an HCV NS5A inhibitor with a potent clinical effect. Nature 465, 96–100 (2010). 20410884 10.1038/nature08960 PMC7094952

[b34] Hou W., Tian Q., Zheng J. & Bonkovsky H. L. Zinc mesoporphyrin induces rapid proteasomal degradation of hepatitis C nonstructural 5A protein in human hepatoma cells. Gastroenterology 138, 1909–1919 (2010). 19909748 10.1053/j.gastro.2009.11.001 PMC2860067

[b35] Mizushima N., Yoshimori T. & Levine B. Methods in mammalian autophagy research. Cell 140, 313–326 (2010). 20144757 10.1016/j.cell.2010.01.028 PMC2852113

[b36] Brass V. *et al.* An amino-terminal amphipathic alpha-helix mediates membrane association of the hepatitis C virus nonstructural protein 5A. J. Biol. Chem. 277, 8130–8139 (2002). 11744739 10.1074/jbc.M111289200

[b37] Kabeya Y. *et al.* LC3, a mammalian homologue of yeast Apg8p, is localized in autophagosome membranes after processing. EMBO J. 19, 5720–5728 (2000). 11060023 10.1093/emboj/19.21.5720 PMC305793

[b38] Huang H. *et al.* Hepatitis C virus inhibits AKT-tuberous sclerosis complex (TSC), the mechanistic target of rapamycin (MTOR) pathway, through endoplasmic reticulum stress to induce autophagy. Autophagy 9, 175–195 (2013). 23169238 10.4161/auto.22791 PMC3552882

[b39] Shinohara Y. *et al.* Unfolded protein response pathways regulate Hepatitis C virus replication via modulation of autophagy. Biochem. Biophys. Res. Commun. 432, 326–332 (2013). 23395875 10.1016/j.bbrc.2013.01.103 PMC7124205

[b40] Yamamoto A. *et al.* Bafilomycin A1 prevents maturation of autophagic vacuoles by inhibiting fusion between autophagosomes and lysosomes in rat hepatoma cell line, H-4-II-E cells. Cell Struct. Funct. 23, 33–42 (1998). 9639028 10.1247/csf.23.33

[b41] Richetta C. *et al.* Sustained autophagy contributes to measles virus infectivity. PLoS Pathog. 9, e1003599 (2013). 24086130 10.1371/journal.ppat.1003599 PMC3784470

[b42] Bjorkoy G. *et al.* p62/SQSTM1 forms protein aggregates degraded by autophagy and has a protective effect on huntingtin-induced cell death. J. Cell Biol. 171, 603–614 (2005). 16286508 10.1083/jcb.200507002 PMC2171557

[b43] Axe E. L. *et al.* Autophagosome formation from membrane compartments enriched in phosphatidylinositol 3-phosphate and dynamically connected to the endoplasmic reticulum. J. Cell Biol. 182, 685–701 (2008). 18725538 10.1083/jcb.200803137 PMC2518708

[b44] Kimura S., Noda T. & Yoshimori T. Dissection of the autophagosome maturation process by a novel reporter protein, tandem fluorescent-tagged LC3. Autophagy 3, 452–460 (2007). 17534139 10.4161/auto.4451

[b45] He Y., Staschke K. A. & Tan S. L. in: Hepatitis C Viruses: Genomes and Molecular Biology ed. Tan S.L.) (2006). 21250377

[b46] Draeby I. *et al.* The calcium binding protein ALG-2 binds and stabilizes Scotin, a p53-inducible gene product localized at the endoplasmic reticulum membrane. Arch. Biochem. Biophys. 467, 87–94 (2007). 17889823 10.1016/j.abb.2007.07.028 PMC2691584

[b47] Dreux M. & Chisari F. V. Impact of the autophagy machinery on hepatitis C virus infection. Viruses 3, 1342–1357 (2011). 21994783 10.3390/v3081342 PMC3185811

[b48] Sung P. S. *et al.* Roles of unphosphorylated ISGF3 in HCV infection and interferon responsiveness. Proc. Natl Acad. Sci. USA 112, 10443–10448 (2015). 26216956 10.1073/pnas.1513341112 PMC4547285

[b49] Der S. D., Zhou A., Williams B. R. & Silverman R. H. Identification of genes differentially regulated by interferon alpha, beta, or gamma using oligonucleotide arrays. Proc. Natl Acad. Sci. USA 95, 15623–15628 (1998). 9861020 10.1073/pnas.95.26.15623 PMC28094

[b50] Oppenheim A. B., Kobiler O., Stavans J., Court D. L. & Adhya S. Switches in bacteriophage lambda development. Annu. Rev. Genet. 39, 409–429 (2005). 16285866 10.1146/annurev.genet.39.073003.113656

[b51] Nikoletopoulou V., Markaki M., Palikaras K. & Tavernarakis N. Crosstalk between apoptosis, necrosis and autophagy. Biochim. Biophys. Acta. 1833, 3448–3459 (2013). 23770045 10.1016/j.bbamcr.2013.06.001

[b52] Pawlotsky J. M. NS5A inhibitors in the treatment of hepatitis C. J. Hepatol. 59, 375–382 (2013). 23567084 10.1016/j.jhep.2013.03.030

[b53] Elazar M. *et al.* Amphipathic helix-dependent localization of NS5A mediates hepatitis C virus RNA replication. J. Virol. 77, 6055–6061 (2003). 12719597 10.1128/JVI.77.10.6055-6061.2003 PMC154017

[b54] Kim M. J. & Yoo J. Y. Inhibition of hepatitis C virus replication by IFN-mediated ISGylation of HCV-NS5A. J. Immunol. 185, 4311–4318 (2010). 20810994 10.4049/jimmunol.1000098

[b55] Sung P. S. *et al.* Hepatitis C virus entry is impaired by claudin-1 downregulation in diacylglycerol acyltransferase-1-deficient cells. J. Virol. 88, 9233–9244 (2014). 24899196 10.1128/JVI.01428-14 PMC4136266

[b56] Lindenbach B. D. *et al.* Complete replication of hepatitis C virus in cell culture. Science 309, 623–626 (2005). 15947137 10.1126/science.1114016

[b57] Wakita T. *et al.* Production of infectious hepatitis C virus in tissue culture from a cloned viral genome. Nat. Med. 11, 791–796 (2005). 15951748 10.1038/nm1268 PMC2918402

[b58] Zhong J. *et al.* Robust hepatitis C virus infection *in vitro*. Proc. Natl Acad. Sci. USA 102, 9294–9299 (2005). 15939869 10.1073/pnas.0503596102 PMC1166622

[b59] Park J. *et al.* Hepatitis C virus infection enhances TNFalpha-induced cell death via suppression of NF-kappaB. Hepatology 56, 831–840 (2012). 22430873 10.1002/hep.25726

[b60] Kang W. & Shin E. C. Colorimetric focus-forming assay with automated focus counting by image analysis for quantification of infectious hepatitis C virions. PLoS ONE 7, e43960 (2012). 22937136 10.1371/journal.pone.0043960 PMC3427175

