# Supplementary material for: Interferon-inducible protein SCOTIN interferes with HCV replication through the autolysosomal degradation of NS5A
Source: Nat Commun. 2016 Feb 12;7:10631. doi: 10.1038/ncomms10631 (PMC4754343; doi:10.1038/ncomms10631)
Supplement: Supplementary Information — Supplementary Figures 1-7 and Supplementary Tables 1-3 [file ncomms10631-s1.pdf]

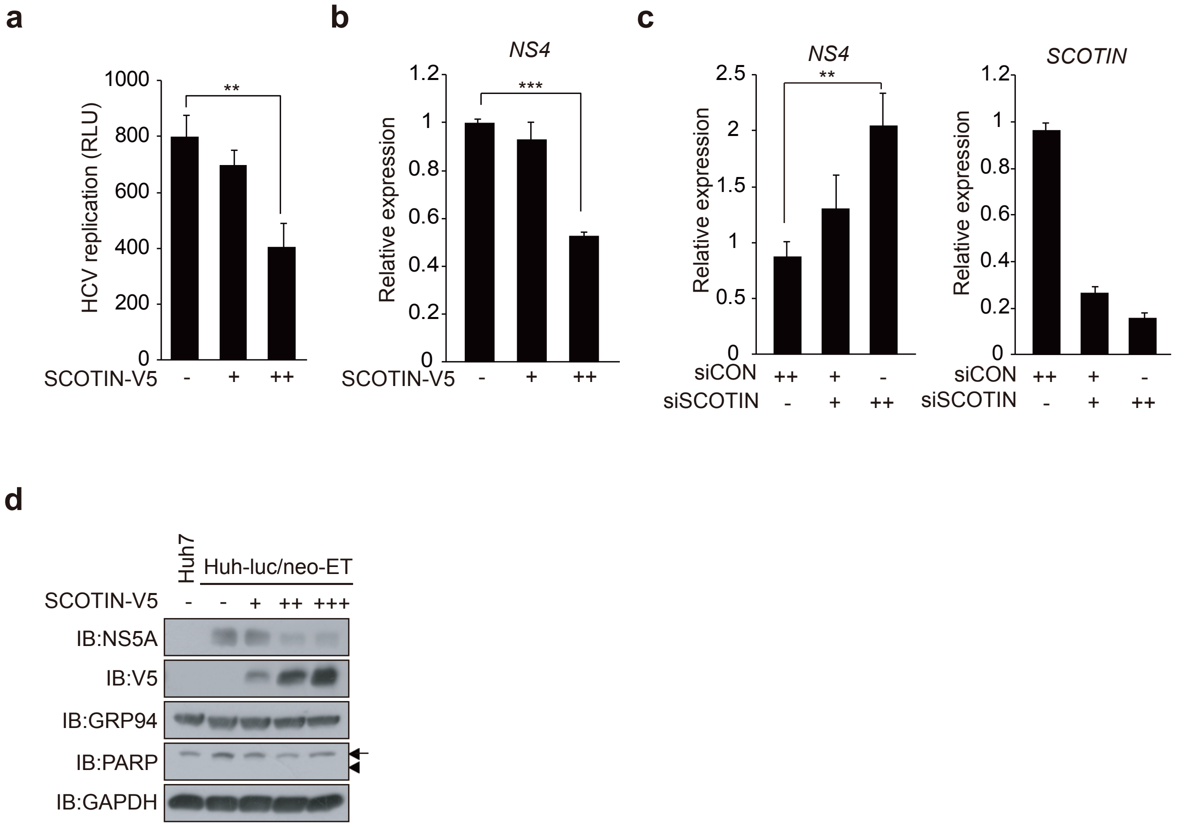

Supplementary Figure 1. SCOTIN restricts HCV replication (a-b) Huh-luc/neo-ET replicon cells (a) or Huh-neo-5-15 replicon cells (b) were transfected with an empty (pDEST-51) or SCOTIN-V5 vector, and the HCV replication levels were measured by a luciferase assay (a) or by RT-qPCR of HCV NS4 (b). (c) Huh-neo-5-15 cells were transfected with control or SCOTIN siRNA, and the HCV NS4 RNA (left) or *SCOTIN* mRNA (right) levels were assessed by RT-qPCR. (a-c) The bars indicate the mean value ± s.d. obtained from three experiments. (d) Huh-neo-5-15 replicon cells were transfected with varying amounts of SCOTIN plasmid, and total lysates were analyzed by immunoblotting. To promote a gradual increase in SCOTIN-V5 expression, cells were transfected with 0.5 μg (+), 1 μg (++), or 3 μg (+++) of plasmid. The arrow and arrowhead indicate the sizes of the full-length PARP-1 and cleaved PARP-1 proteins, respectively.

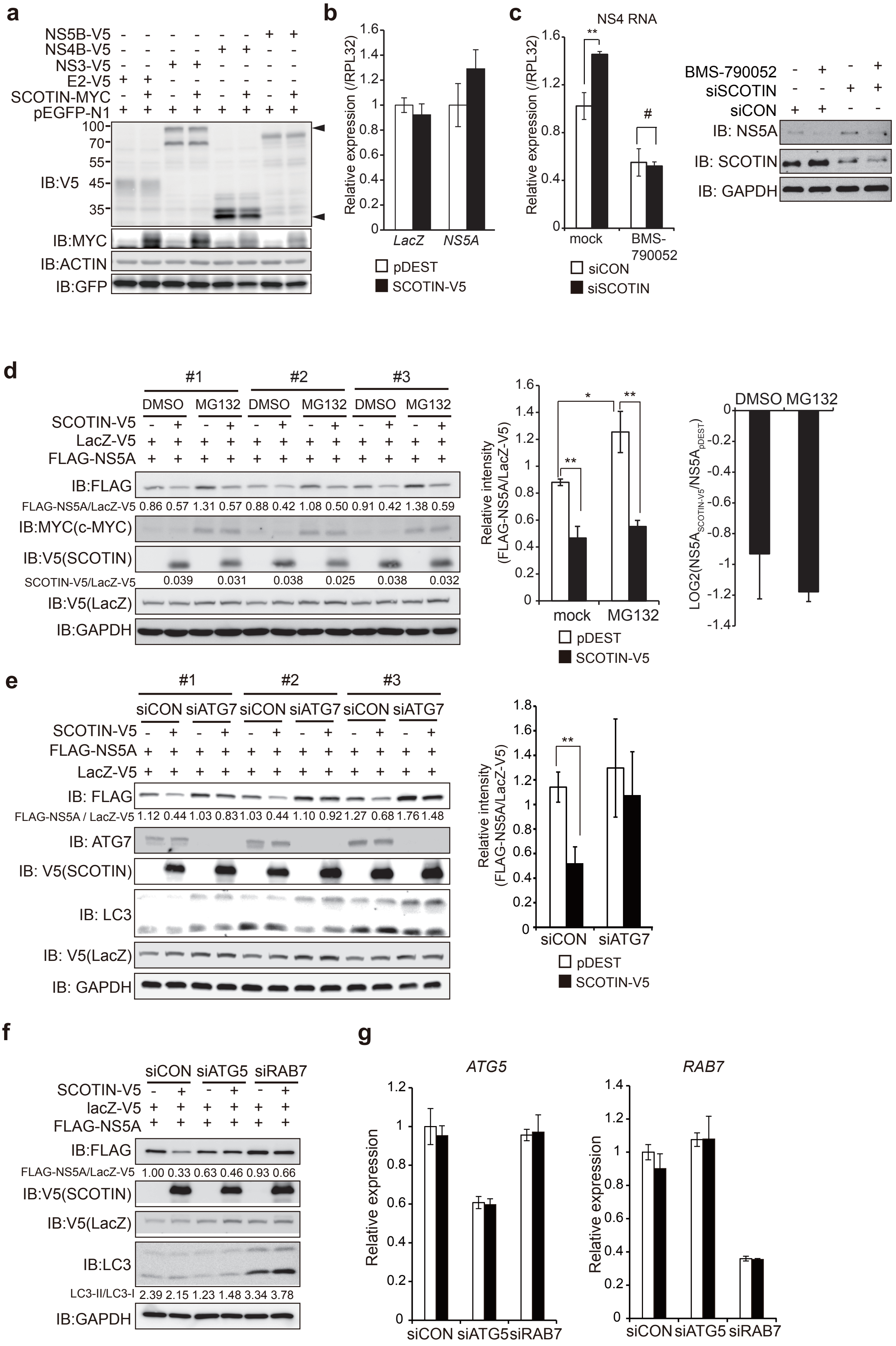
a
NS5B-V5
NS4B-V5
NS3-V5
E2-V5
SCOTIN-MYC
pEGFP-N1
100
70
55
45
35
IB:V5
IB:MYC
IB:ACTIN
IB:GFP
b
Relative expression (/RPL32)
LacZ
NS5A
pDEST
SCOTIN-V5
c
NS4 RNA
Relative expression (/RPL32)
mock
BMS-790052
siCON
siSCOTIN
BMS-790052
siSCOTIN
siCON
IB: NS5A
IB: SCOTIN
IB: GAPDH
d
#1
#2
#3
DMSO
MG132
SCOTIN-V5
LacZ-V5
FLAG-NS5A
IB:FLAG
FLAG-NS5A/LacZ-V5 0.86 0.57 1.31 0.57 0.88 0.42 1.08 0.50 0.91 0.42 1.38 0.59
IB:MYC(c-MYC)
IB:V5(SCOTIN)
SCOTIN-V5/LacZ-V5 0.039 0.031 0.038 0.025 0.038 0.032
IB:V5(LacZ)
IB:GAPDH
Relative intensity (FLAG-NS5A/LacZ-V5)
mock
MG132
pDEST
SCOTIN-V5
DMSO MG132
LOG2(NS5A SCOTIN-V5/NS5A pDEST)
e
#1
#2
#3
siCON
siATG7
SCOTIN-V5
FLAG-NS5A
LacZ-V5
IB: FLAG
FLAG-NS5A / LacZ-V5 1.12 0.44 1.03 0.83 1.03 0.44 1.10 0.92 1.27 0.68 1.76 1.48
IB: ATG7
IB: V5(SCOTIN)
IB: LC3
IB: V5(LacZ)
IB: GAPDH
Relative intensity (FLAG-NS5A/LacZ-V5)
siCON
siATG7
pDEST
SCOTIN-V5
f
siCON
siATG5
siRAB7
SCOTIN-V5
lacZ-V5
FLAG-NS5A
IB:FLAG
FLAG-NS5A/LacZ-V5 1.00 0.33 0.63 0.46 0.93 0.66
IB:V5(SCOTIN)
IB:V5(LacZ)
IB:LC3
LC3-II/LC3-I 2.39 2.15 1.23 1.48 3.34 3.78
IB:GAPDH
g
ATG5
RAB7
Relative expression
siCON
siATG5
siRAB7

(continuation)

Supplementary Figure 2. SCOTIN promotes NS5A degradation via autophagy (a) Huh-7 cells were transfected with the indicated plasmids or with siRNAs for 48 hrs, and total cell lysates were subjected to immunoblotting using the indicated antibodies. GFP-expressing pEGFP-N1 plasmids were included to monitor transfection efficiency. The expected sizes of NS3 and NS4B are indicated. (b) Huh-7 cells were transfected with LacZ-V5 and FLAG-NS5A along with an empty or SCOTIN-V5-expressing vector. RNA extraction was performed in triplicate, and RT-qPCR was conducted to assess LacZ and NS5A expression. (c) Huh-neo-5-15 cells were transfected with control or SCOTIN siRNA, followed by treatment with BMS790052 (1 nM) for 24 hrs. Cell lysates were subjected to immunoblotting with the indicated antibodies, and RT-qPCR was performed to measure the HCV NS4 RNA levels. (d) (left)Triplicate experiment as in Fig. 2c. (middle)The mean and s.d. of the level of FLAG-NS5A/LacZ-V5 from triplicate experiments. (right)Reduction rate by SCOTIN overexpression compared with control was shown. (e) (left)Triplicate experiments as in Fig. 2g. (right)The mean and s.d. of the value of FLAG-NS5A/LacZ-V5 from triplicate experiments. (f-g) Huh-7 cells were transfected with the indicated plasmids or with siRNAs for 48 hrs. (f) Total cell lysates were subjected to immunoblotting using the indicated antibodies. (g) RNA levels were analyzed by RT-qPCR using primers targeting *ATG5* or *RAB7* and normalized to the *RPL32* level. Each graph represents the mean value of triplicate experiments, and the error bar indicates the s.d.. The asterisks indicate the p-values calculated using the t-test. *: p-value<0.05, **: p-value<0.01, ***: p-value<0.001, #: “not significant”

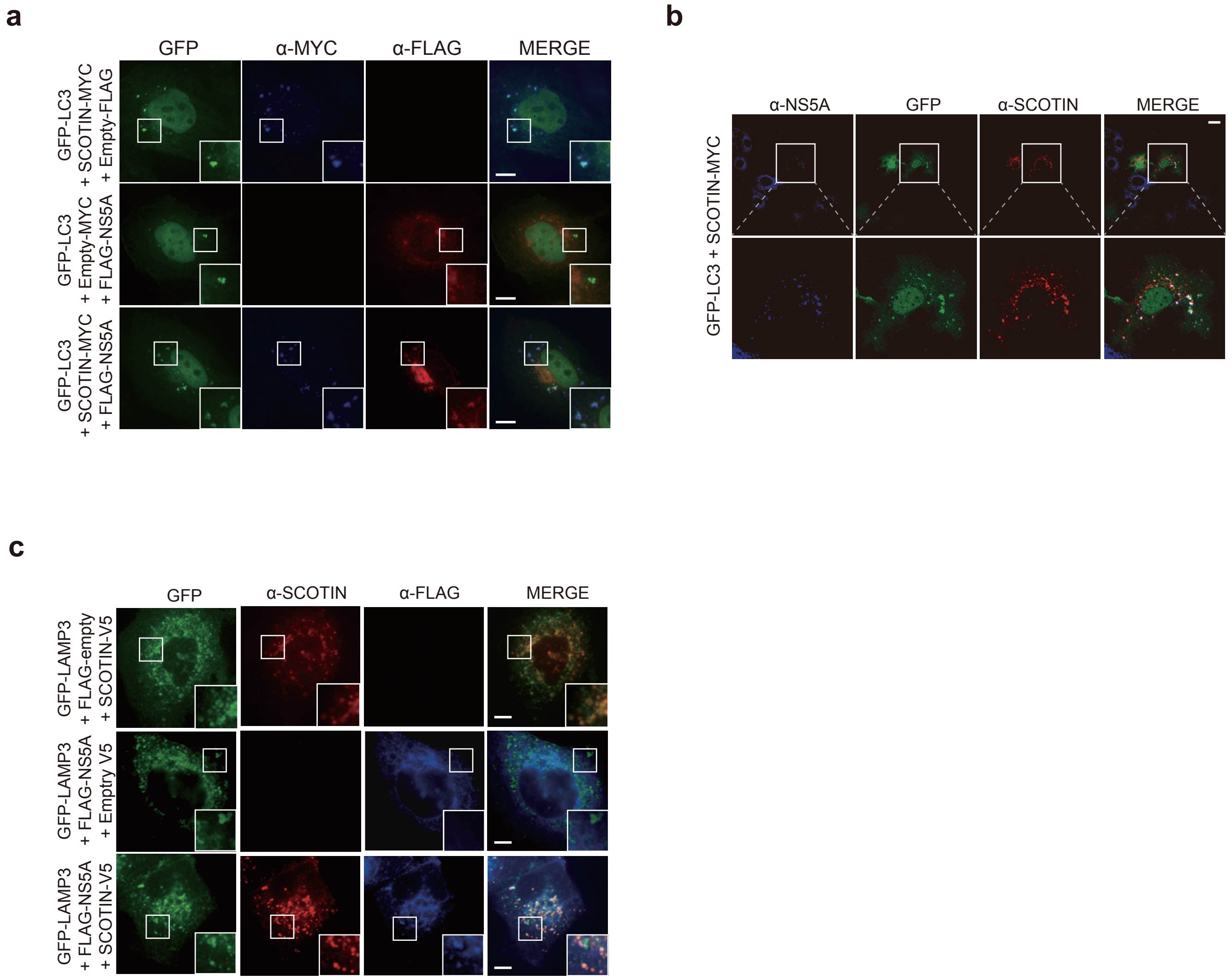

Supplementary Figure 3. SCOTIN promotes NS5A trafficking to autophagosomes (a) Huh-7 cells were transfected with the indicated plasmids. Fluorescence immunostaining analyses were performed to detect FLAG-NS5A (red), SCOTIN-MYC (blue), and GFP-LC3 (green) using FLAG and MYC antibodies. Scale bar, 10μm. (b) Huh-neo-5-15 replicon cells were transfected with GFP-LC3 and SCOTIN-MYC plasmids. A confocal fluorescence image was obtained, with detection of NS5A (blue), SCOTIN-MYC (red), and GFP-LC3 (green) signals. Scale bar, 20μm. (c) Huh-7 cells were transfected with the indicated plasmids, followed by CQ (50 μM) treatment for 6 hrs. They were then immunostained using SCOTIN and FLAG antibodies. Fluorescence microscopy was performed to detect FLAG-NS5A (blue) and SCOTIN-V5 (red) with GFP-LAMP1 (green) after immunostaining with an anti-SCOTIN or anti-FLAG antibody. Scale bar, 10μm. Enlarged images of the small box inside of each figure are presented at the right bottom corner.

a

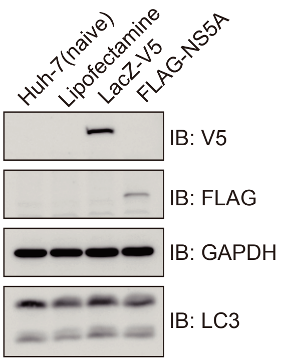

b

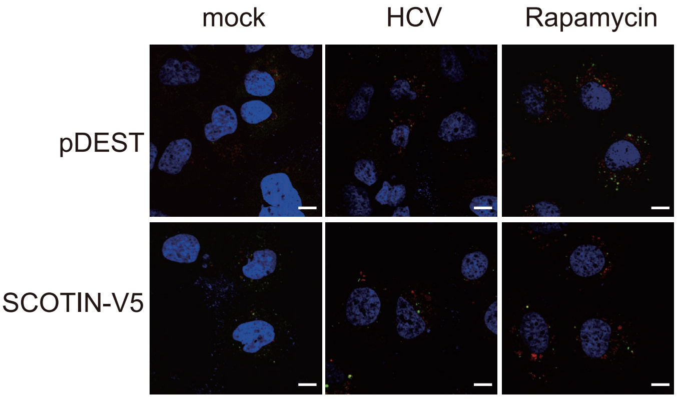

Supplementary Figure 4. Overexpression of NS5A or SCOTIN does not alter overall autophagy flux (a) Huh-7 cells were transfected with indicated plasmids and cell lysates were subjected to immunoblotting using indicated antibodies. (b)Representative images of Fig. 4e. Scale bars, 10μm.

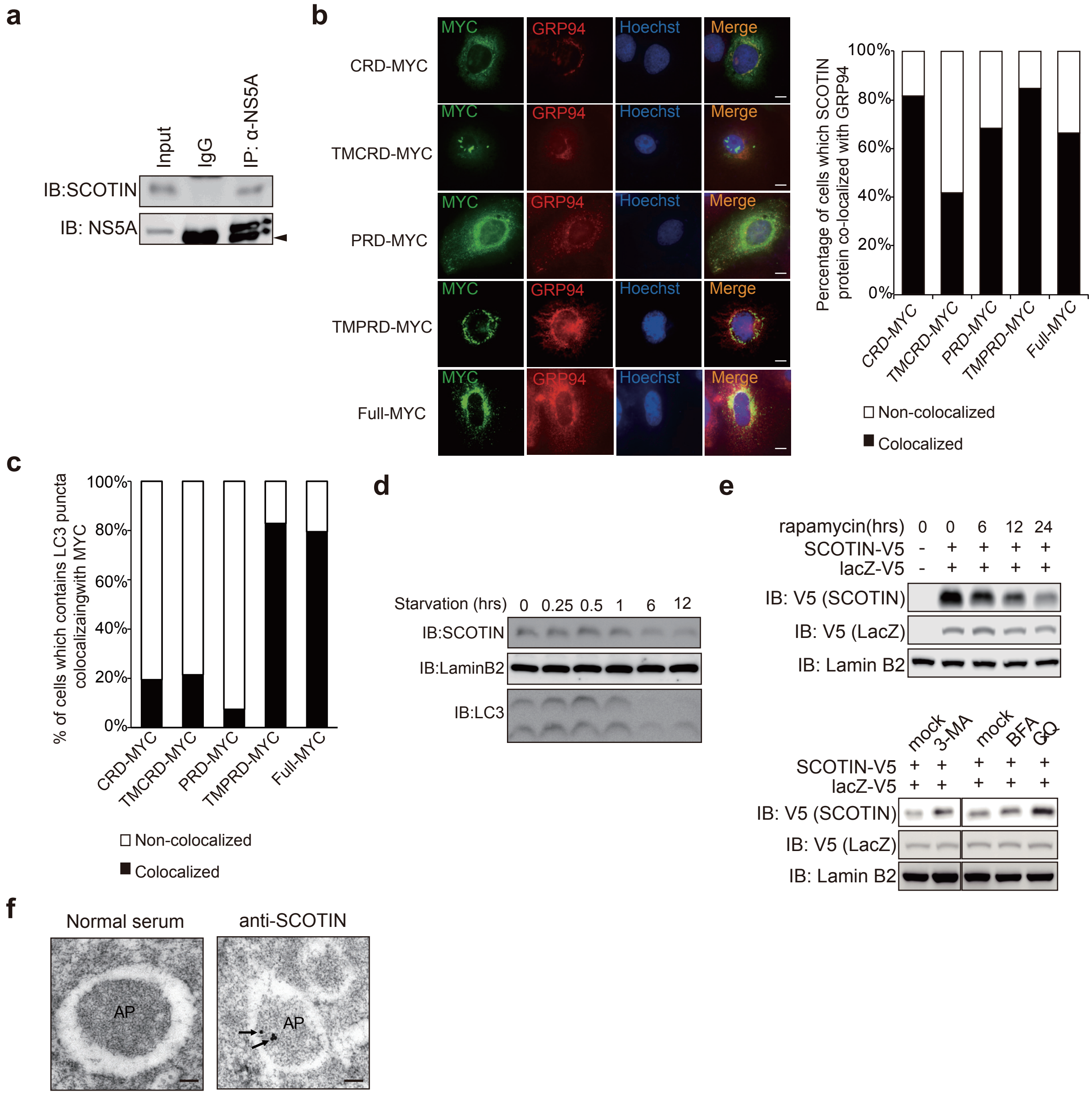

Supplementary Figure 5. SCOTIN is a substrate of autophagy (a) The NS5A protein was immunoprecipitated from Huh-neo-5-15 replicon cell lysates using an NS5A antibody, and the interacting proteins were analyzed by immunoblotting. The arrowhead indicates the heavy chain of the antibody. (b) Huh-7 cells were transfected with the indicated SCOTIN constructs, and immunostaining was performed using antibodies against GRP94 and MYC, followed by nuclear staining with Hoechst. Fluorescence microscopy analysis was performed to detect MYC (green), GRP94 (red), and Hoechst (blue). (left) Representative fluorescence microscopy images are shown. (right) The percentage of cells containing MYC co-localized with GRP94 is shown (N=20). Scale bars, 10µm. (c) Huh-7 cells were transfected with the indicated constructs, and immunofluorescence analysis was performed using LC3 and MYC antibodies, followed by Hoechst staining. Fluorescence microscopy analysis was performed to detect LC3 (green), MYC (red), and Hoechst (blue), and representative images are shown in Fig. 5f. For statistical analysis, the percentage of cells that contained co-localized with LC3 puncta was counted from each set (N=36 for CRD, N=28 for TMCRD, N=27 for PRD, N=35 for TMPRD, and N=39 for Full). (d) Huh-7 cells were incubated in starvation media for the indicated durations, and cell lysates were subjected to immunoblotting using the indicated antibodies. (e) Western blot analysis of transiently transfected with SCOTIN-V5 and LacZ-V5 and then treated with rapamycin(2µM) for the indicated durations (top) or with 3-MA (10 mM), BFA (100 µM) or CQ (50 µM) for 12 hrs (bottom). (f) Huh-7 cells were infected with HCVcc (10 MOI) for 3 days and subjected to immunogold electron microscopy using a SCOTIN antibody. (left) Normal serum in place of SCOTIN antibody was used as a negative control. (right) The gold particles (30-nm, indicated by the arrows) show the SCOTIN protein in an autophagosome (AP).

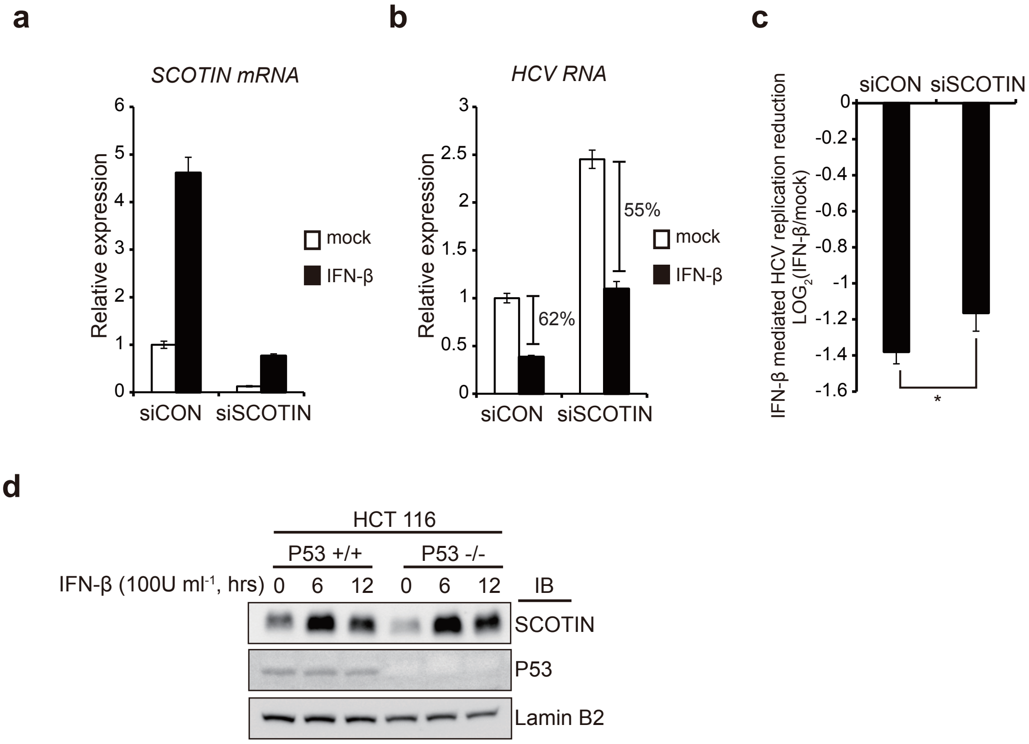

Supplementary Figure 6. SCOTIN contributes to the antiviral activity of interferon-β against HCV (a-c) Huh-neo-5-15 cells were transfected with control or SCOTIN siRNA, followed by treatment with IFN-β (100U ml^{-1}) for 12 hrs. RNA was extracted from cells and RT-qPCR was performed to measure SCOTIN mRNA (a) or the HCV RNA level (b). (c) Reduced HCV RNA level when IFN-β was treated compared to control was shown. Each graph represents the mean value of triplicate experiments, and the error bar indicates the s.d.. The asterisks indicate the p-values calculated using the t-test. *: p-value<0.05. (d)HCT116 ($p53^{+/+}$) and HCT116 ($p53^{-/-}$) cells were treated with IFN-β (100 U ml^{-1}) for the indicated durations. Protein lysates were extracted and analyzed by immunoblotting with the indicated antibodies.

Fig. 1b

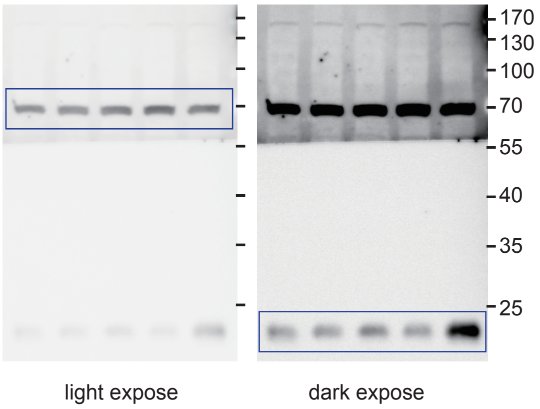

Fig. 1e

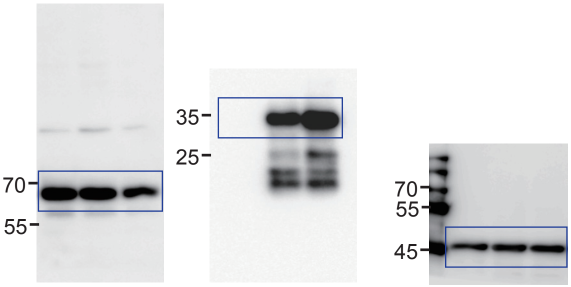

Fig. 1h

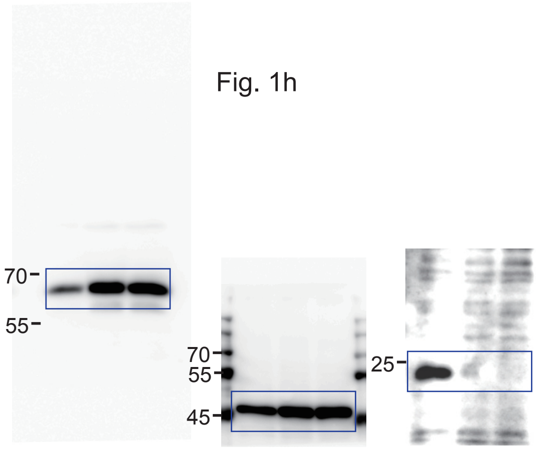

Fig. 2a

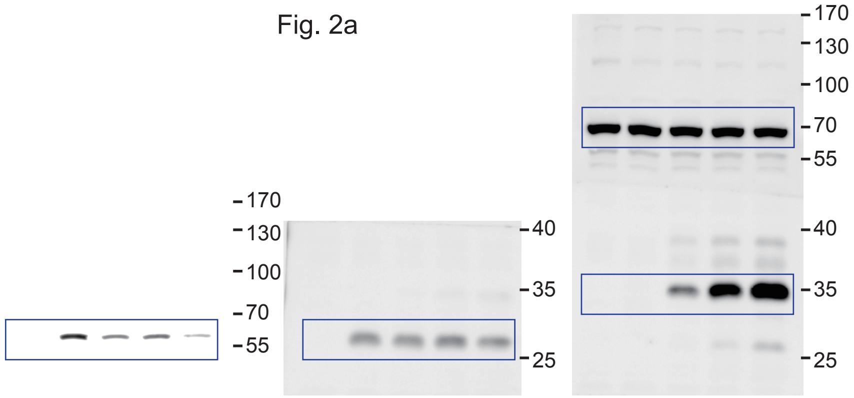

Fig. 2b

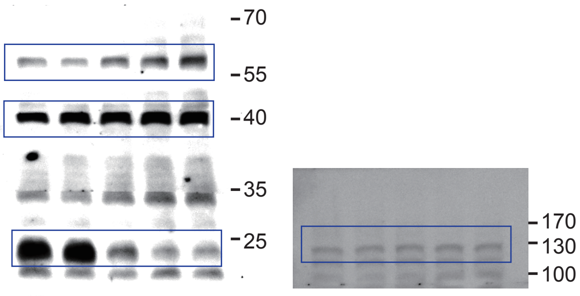

Fig. 2c

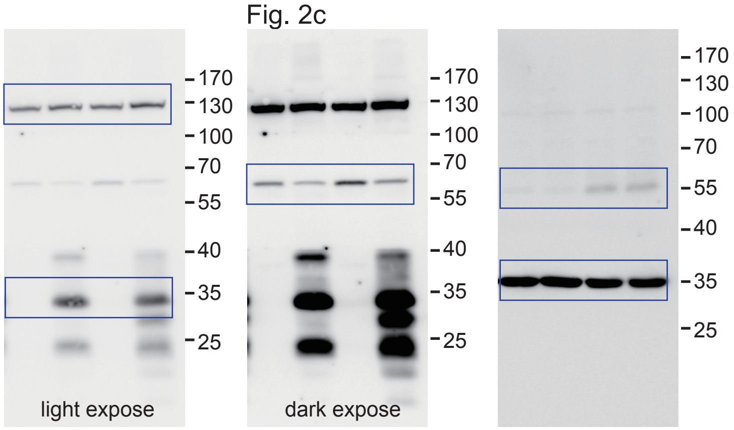

Fig. 2d

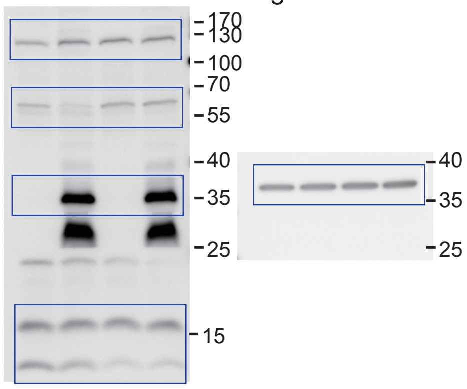

Fig. 2e-f

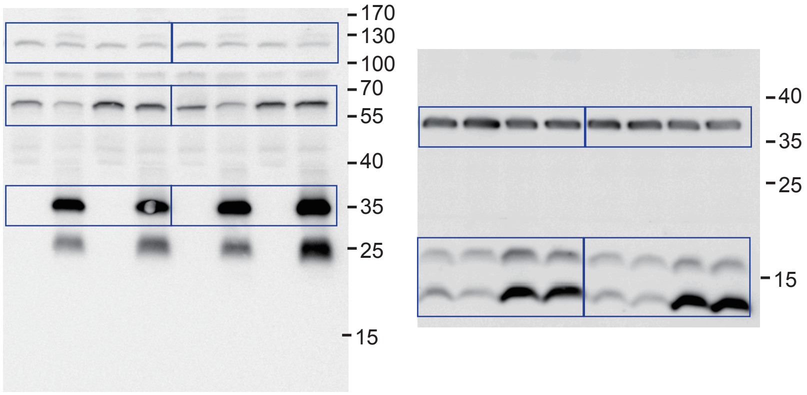

Supplementary Figure 7. Uncropped images of blots

Fig. 2g

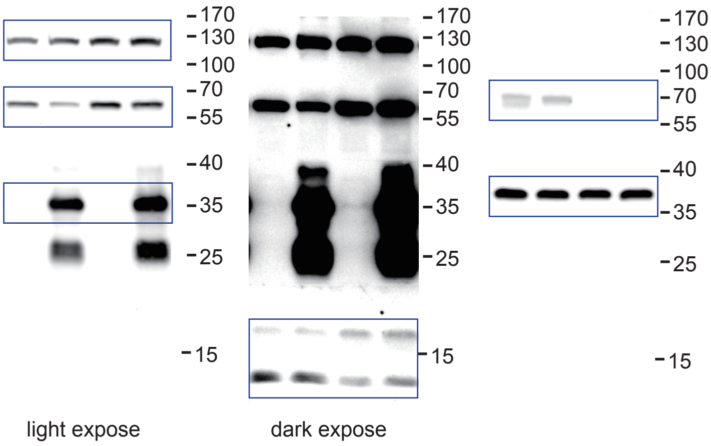

Fig. 2i

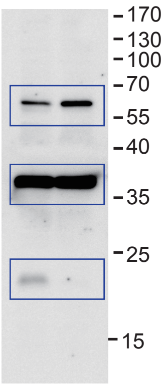

Fig. 3a

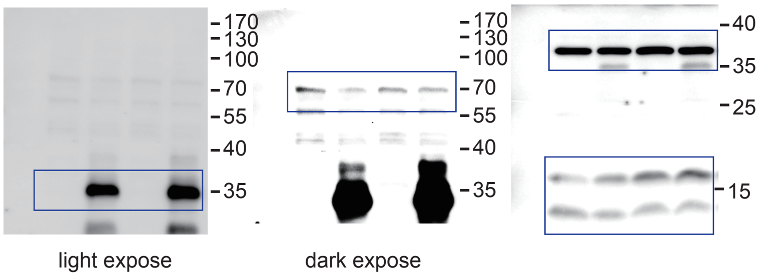

Fig. 3b

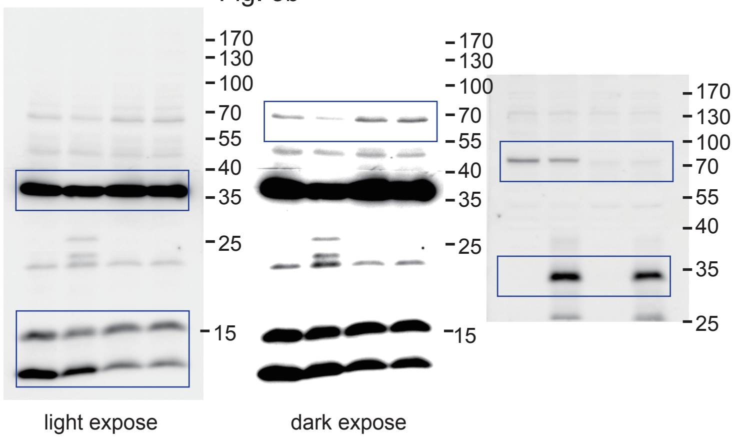

Fig. 3c

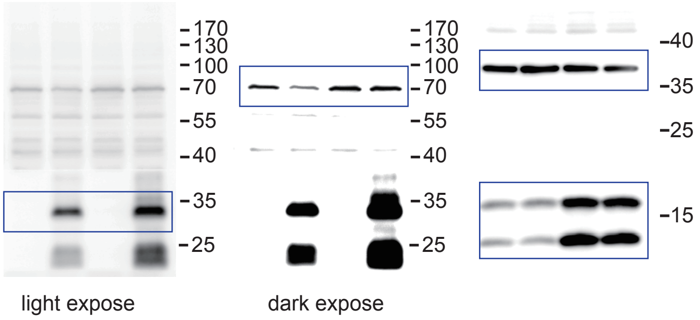

Fig. 3d

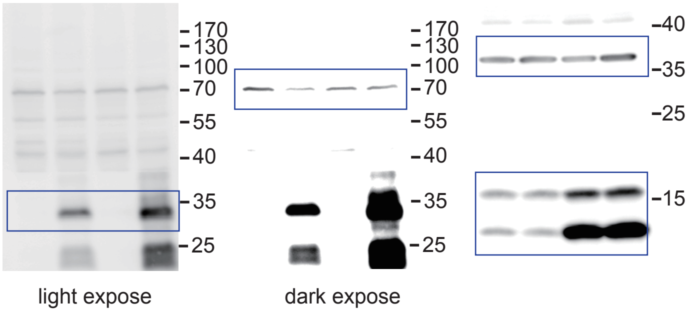

Fig. 3f

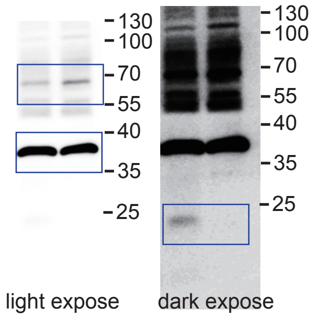

Fig. 4a

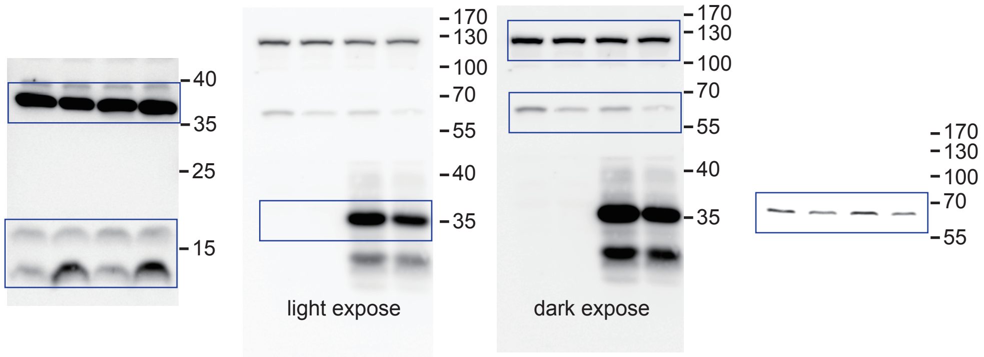

Supplementary Figure 7. Uncropped images of blots (continuation)

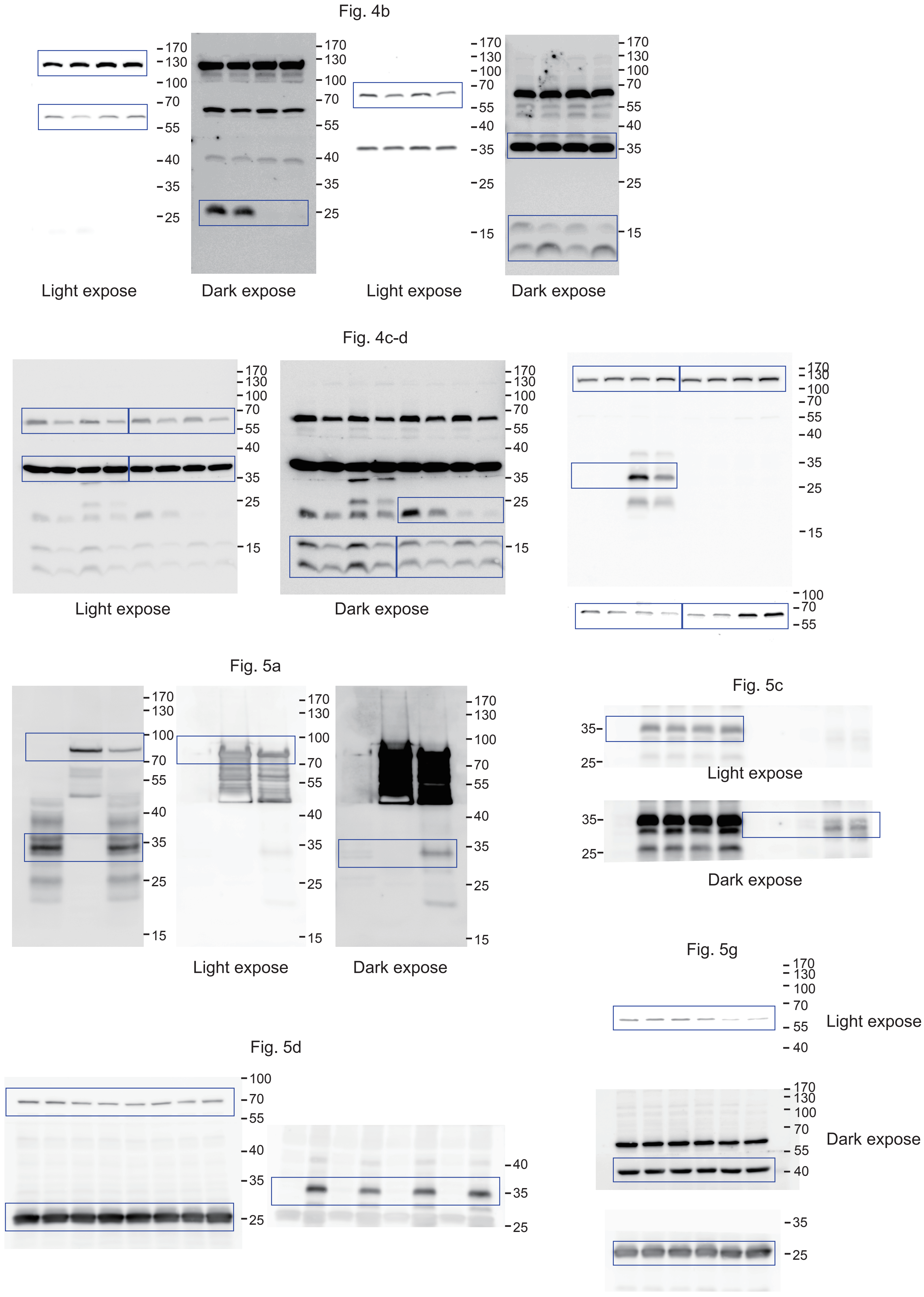

Supplementary Figure 7. Uncropped images of blots (continuation)

Fig. 5h

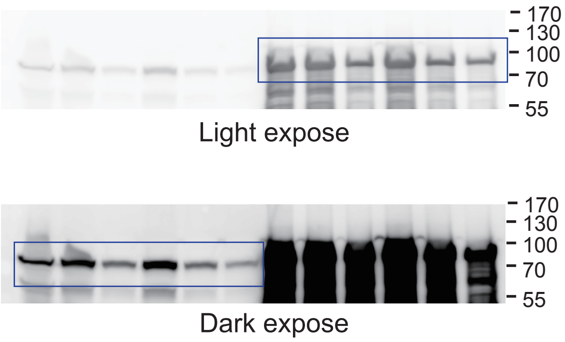

Fig. 5j

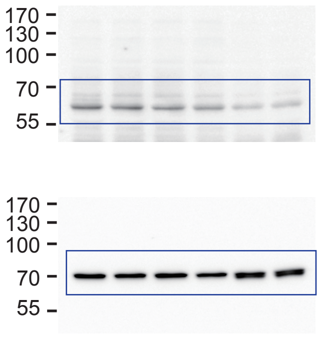

Fig. 6a

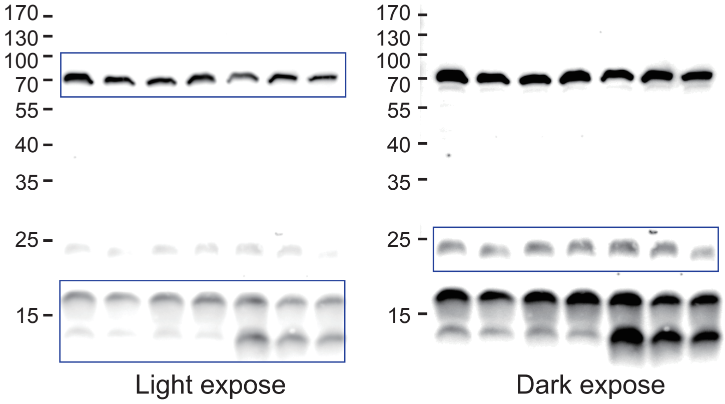

Fig. 6b

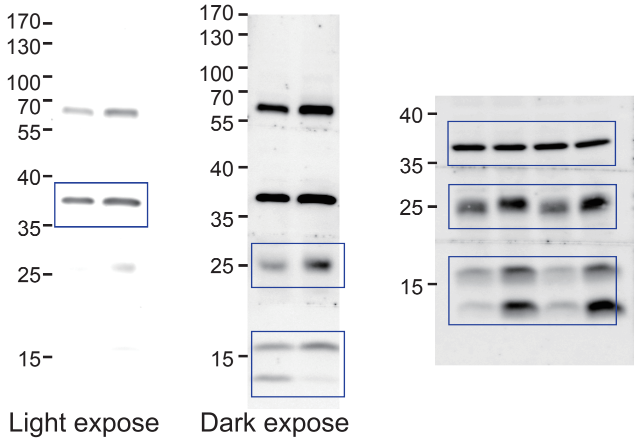

Fig. 6e

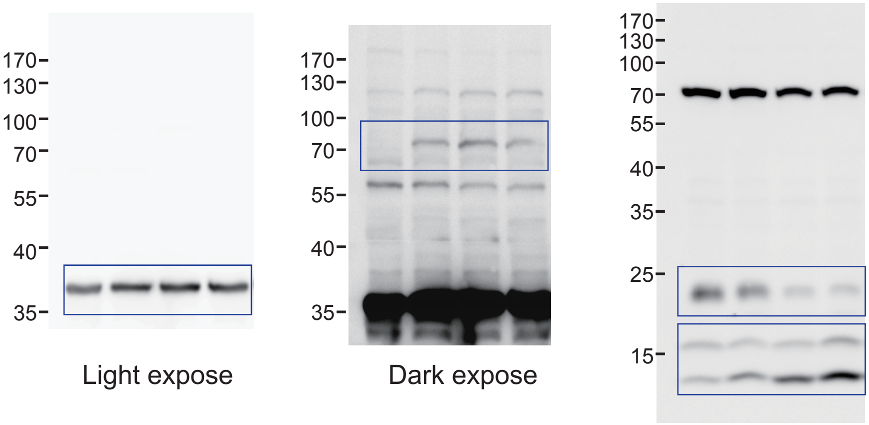

Supplementary Figure 7. Uncropped images of blots (continuation)

Name	Sense sequence (5' → 3')
ATG7	CCAACACACUCGAGUCUUUTT
SCOTIN #1	GUACCUGUGAUGACCAAUATT
SCOTIN #2	GAGCUGUCUUAGCUCAAAUTT
SCOTIN #3	GGUCUGUACACUUGUUUAUTT
ATG5	GCAACUCUGGAUGGGAUUGT
RAB7	GGAUGACCUCUAGGAAGAATT
Control	UUCUCCGAACGUGUCACGUUU

Supplementary Table 1. Sequences of siRNAs

**Primers**	**Oligomer sequences (5' → 3')**
pEBG_NS5A_BamHI_F	5’-CGCGGATTCTCCGGCTCGTGGCTAAGG-3’
pEBG_NS5A_Full_ClaI_R	5’-CCATCGATCTAGCAGCAGACGACGTCCT-3’
pEBG_NS5A_D1_ClaI_R	5’- CCATCGATCTACGTCTCTGCTGTAATGTG-3’
pEBG_NS5A_D1+D2_ClaI_R	5’-CCATCGATCTAGCATCCGTGTACTACCGG-3’
pDEST-V5_SCOTIN_SpeI_F	5'-GTCACTAGTGCCATGGCTGCGCCGGCG-3'
pDEST-V5_SCOTIN_NheI_R	5'-GCAGCTAGCGGGAATTGTCTTTAGGGA-3'
pcDNA_SCOTIN_MYC_F	5'-CGGGATCCATGGCTGCGCCGGCGCCC-3'
pcDNA_SCOTIN_MYC_R	5'-GCTCTAGAGGGAATTGTCTTTAGGGAATCCAT-3'
pcDNA_CRD_MYC_R	5'-GCTCTAGATGACATAGGGTCACTGTCAAAAC-3'
pcDNA_TMCRD_MYC_R	5'-GCTCTAGAGAAGCAGATGATGATAGTGGCAA-3'
pcDNA_PRD_MYC_F(ΔTMCRD)	5'-CTGCCGCCGCCTCCGTGCTTCACCTGCTCC-3'
pcDNA_PRD_MYC_R(ΔTMCRD)	5'-GGAGCAGGTGAAGCACGGAGGCGGCGGCAG-3'
pcDNA_TMPRD_MYC_F(ΔCRD)	5'-CTGCCGCCGCCTCCGGGGTTCGGAGCGACC-3'
pcDNA_TMPRD_MYC_R(ΔCRD)	5'-GGTCGCTCCGAACCCCGGAGGCGGCGGCAG-3'

Supplementary Table 2. Sequences of cloning primers

Primers	Sequence
hRPL32_F	5'-AACCCAGAGGCATTGACAAC-3'
hRPL32_R	5'-GTTGCACATCAGCAGCACTT-3'
ACTIN_F	5'-TCATGAAGTGTGACGTTGACATCCGT-3'
ACTIN_R	5'-CCTAGAAGCATTTGCGGTGCACGATG-3'
NS4_F	5'-ACAACAGGCAGCGTGGTCATT-3'
NS4_R	5'-TTCCACATGTGCTTTGCCCA-3'
SCOTIN_F	5'-TGCTGTGGTACCTGTGATGACCAA-3'
SCOTIN_R	5'-AGGAGCAGGTGAAGCAGATGATGA-3'
HCV RNA_F	5'-CGGGAGAGCCATAGTGG-3'
HCV RNA_R	5'-AGTACCACAAGGCCTTTCG-3'
ATG5_F	5'-AGCAACTCTGGATGGGATTG-3'
ATG5_R	5'- AGGTCTTTCAGTCGTTGTCTG -3'
RAB7_F	5'- GGGAAGACATCACTCATGAACC -3'
RAB7_R	5'- TCCTGCTGTGTCCCATATCT -3'
β-ACTIN (for HCV RNA titer measurement)	Taqman primer (appliedbiosystems, 4310881E)

Supplementary Table 3. Sequences of RT-PCR primers
